# Supplementary figures and images for: Dynamic regulation of inter-organelle communication by ubiquitylation controls skeletal muscle development and disease onset
Source: eLife. 2023 Jul 11;12:e81966. doi: 10.7554/eLife.81966 (PMC10356137; doi:10.7554/eLife.81966)

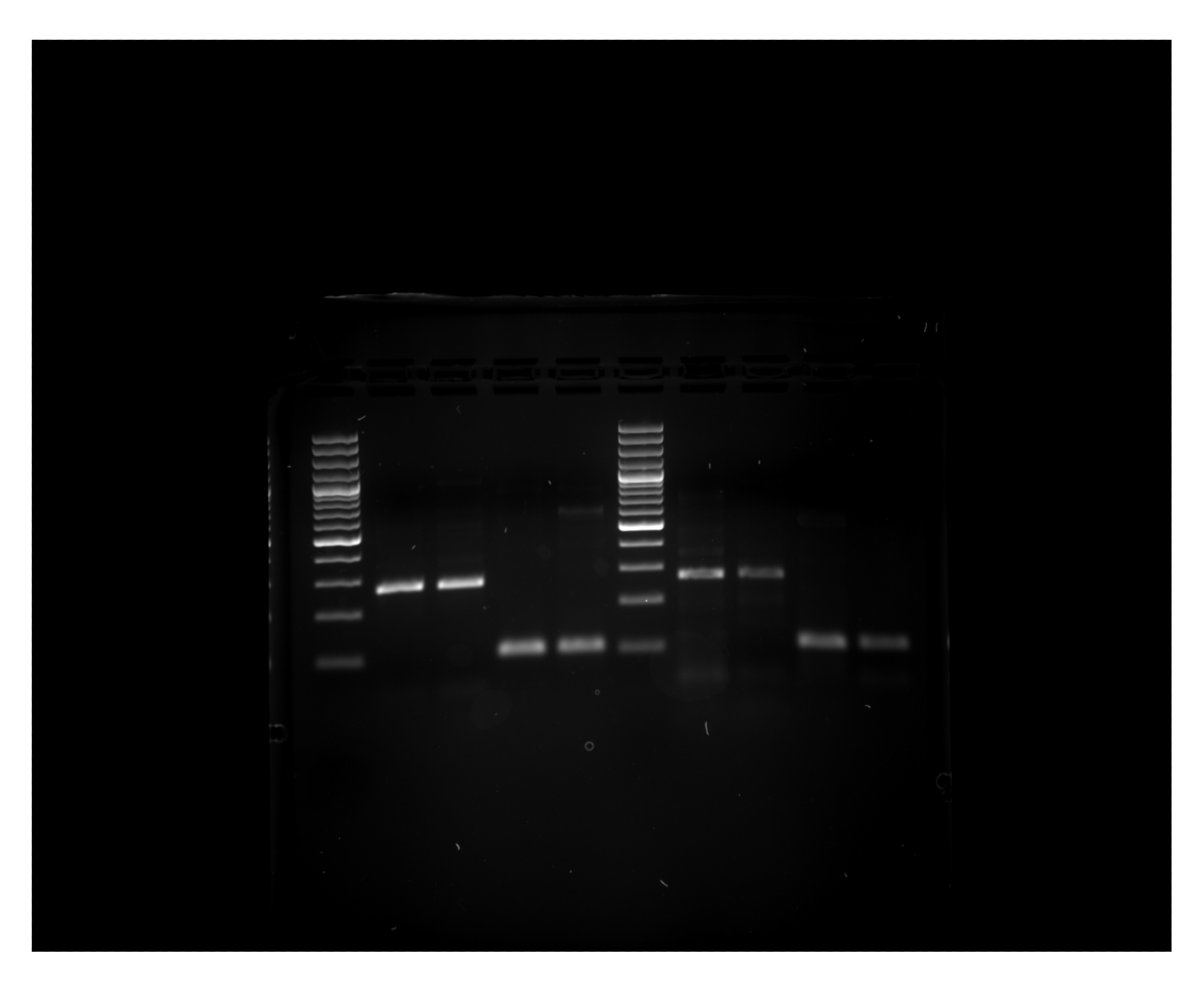

Supplement: Figure 1—figure supplement 1—source data 1. [file elife-81966-fig1-figsupp1-data1.zip › Figure 1-figure supplement 1-Source data 1.tiff]

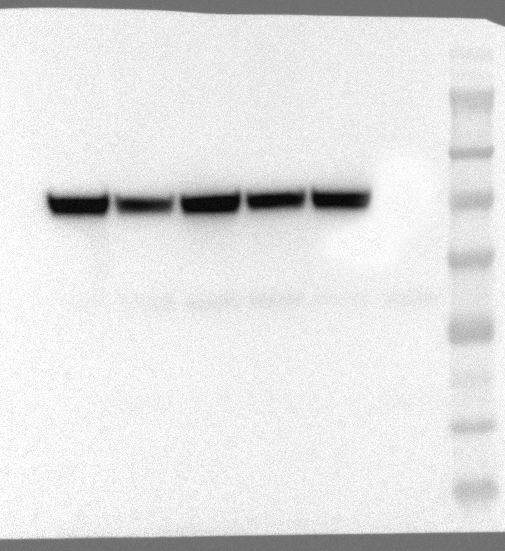

Supplement: Figure 1—figure supplement 1—source data 2. [file elife-81966-fig1-figsupp1-data2.zip › Figure 1-figure supplement 1-Source data 2.tif]

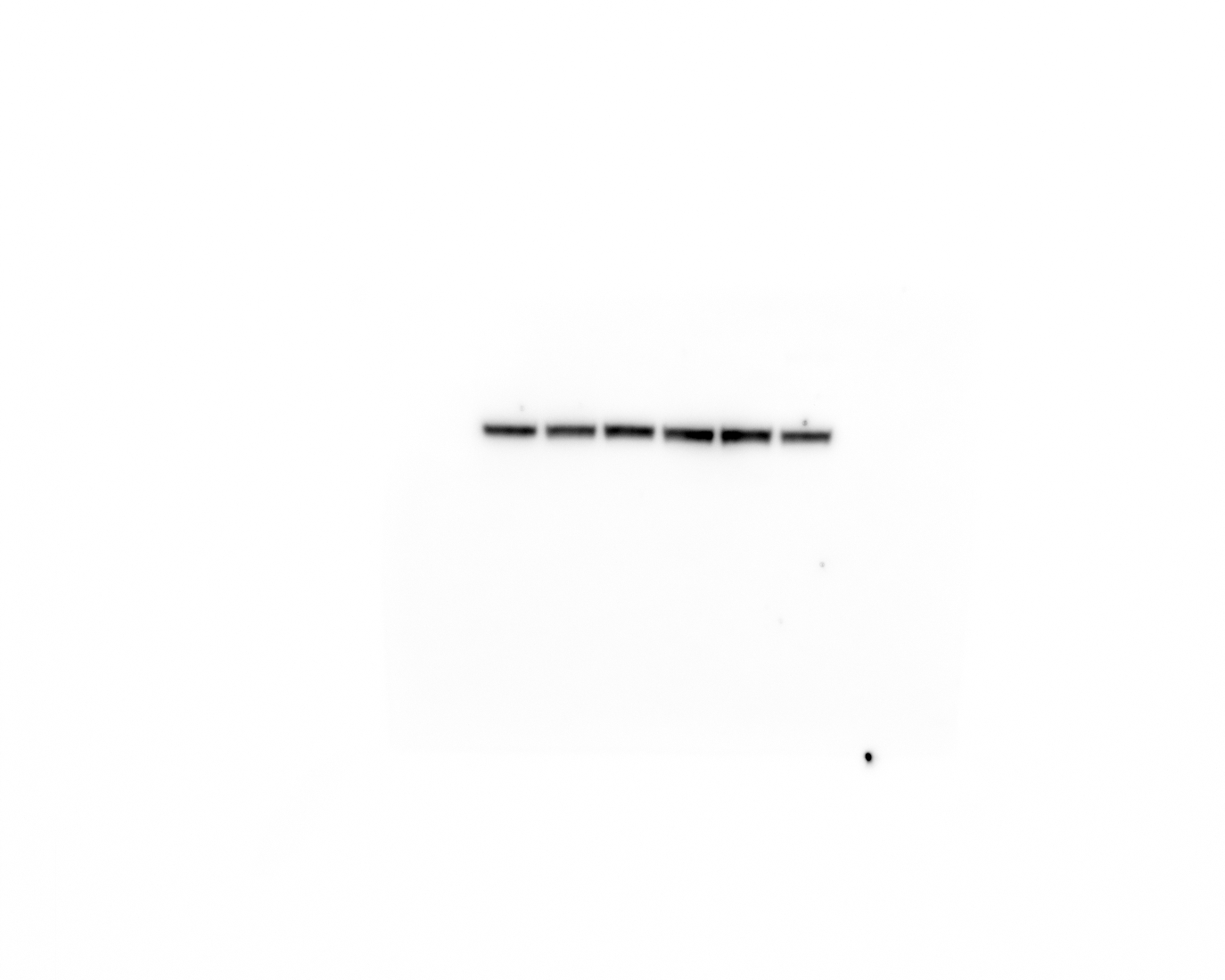

Supplement: Figure 1—figure supplement 1—source data 3. [file elife-81966-fig1-figsupp1-data3.zip › Figure 1-figure supplement 1-Source data 3.tiff]

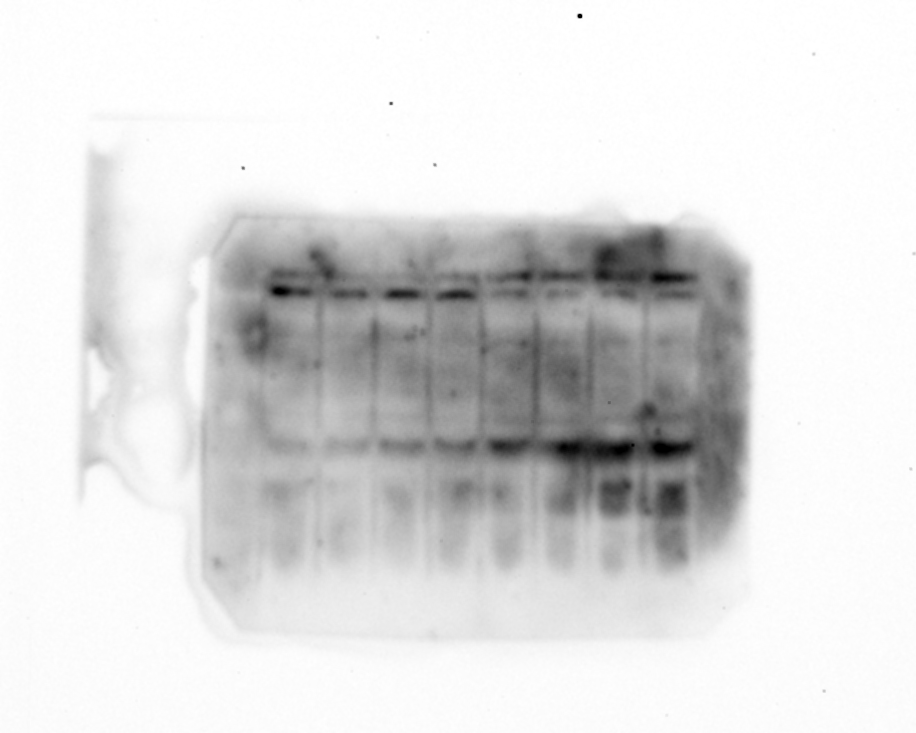

Supplement: Figure 5—source data 1. [file elife-81966-fig5-data1.zip › Figure 5 source data 1.tif]

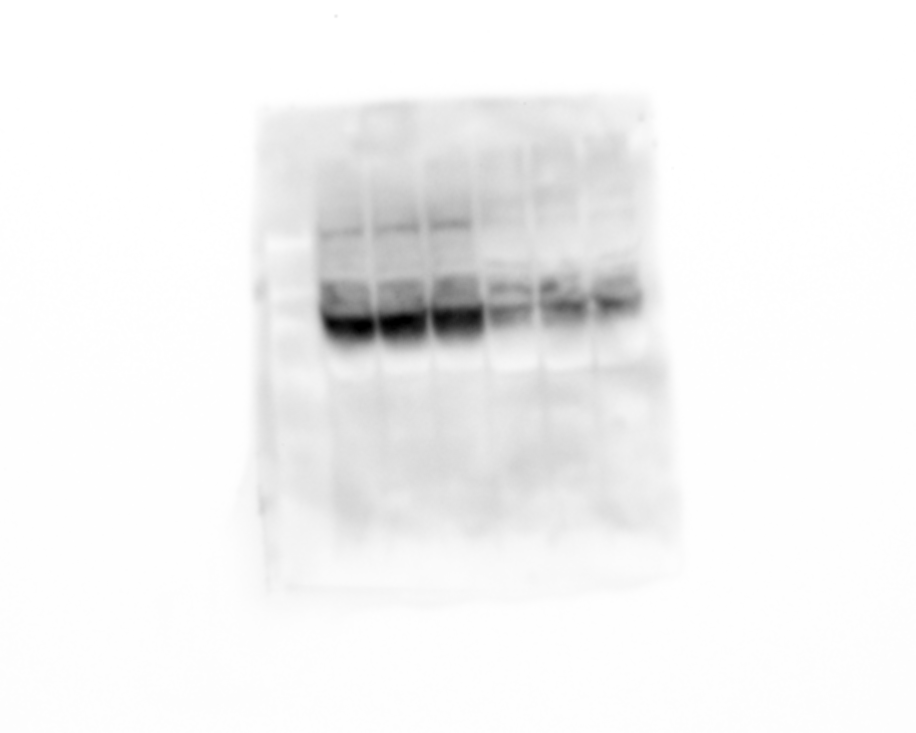

Supplement: Figure 5—source data 2. [file elife-81966-fig5-data2.zip › Figure 5 source data 2.tif]

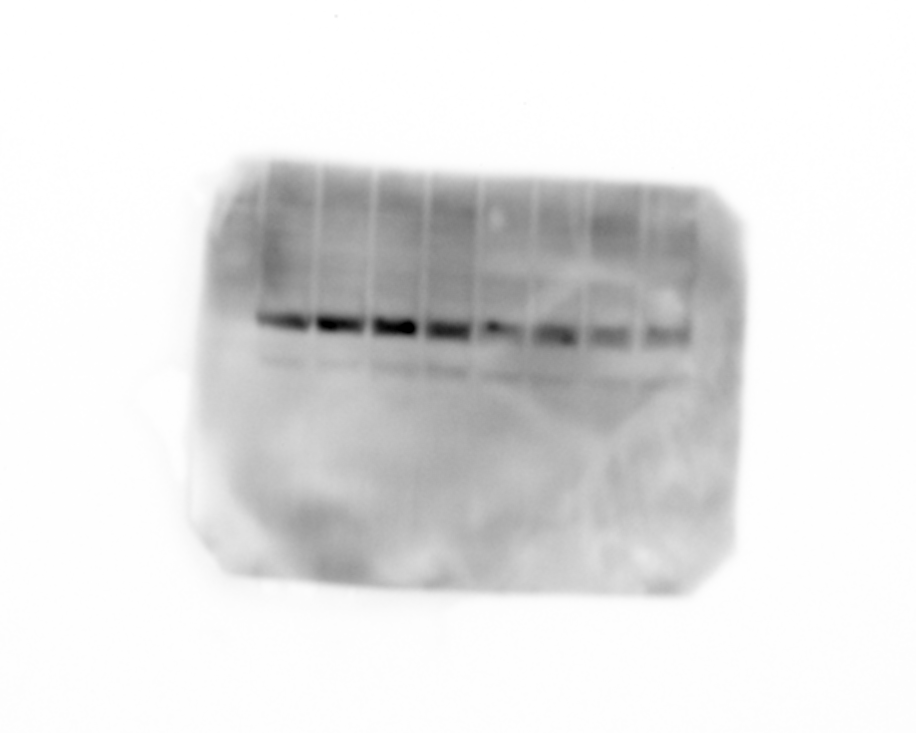

Supplement: Figure 5—source data 3. [file elife-81966-fig5-data3.zip › Figure 5 source data 3.tiff]

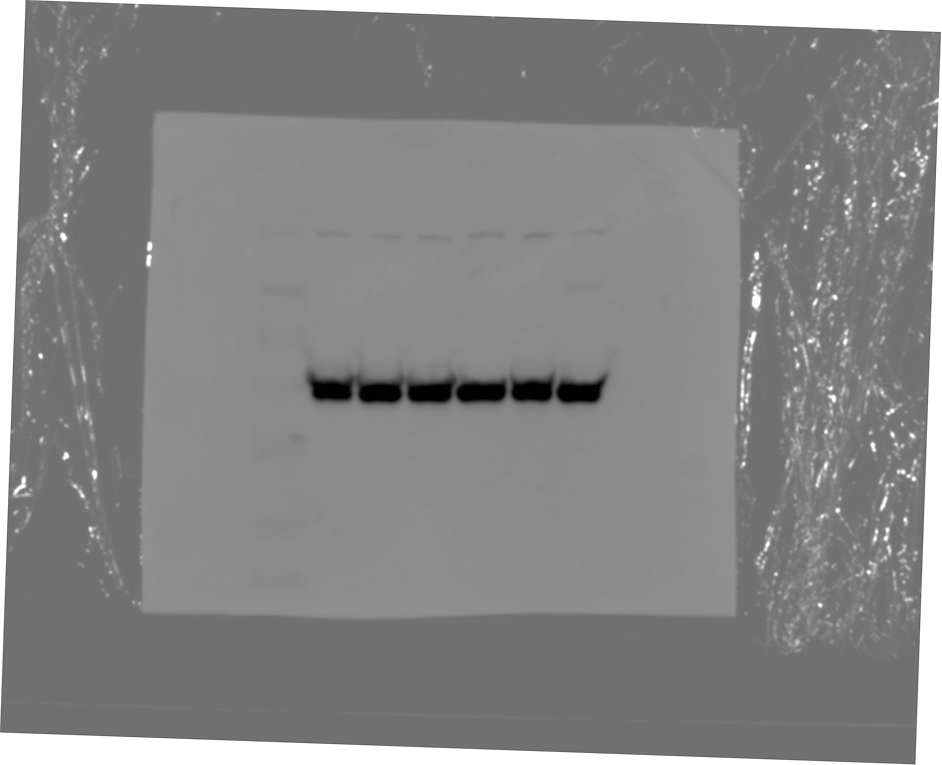

Supplement: Figure 5—source data 4. [file elife-81966-fig5-data4.zip › Figure 5 source data 4.tif]

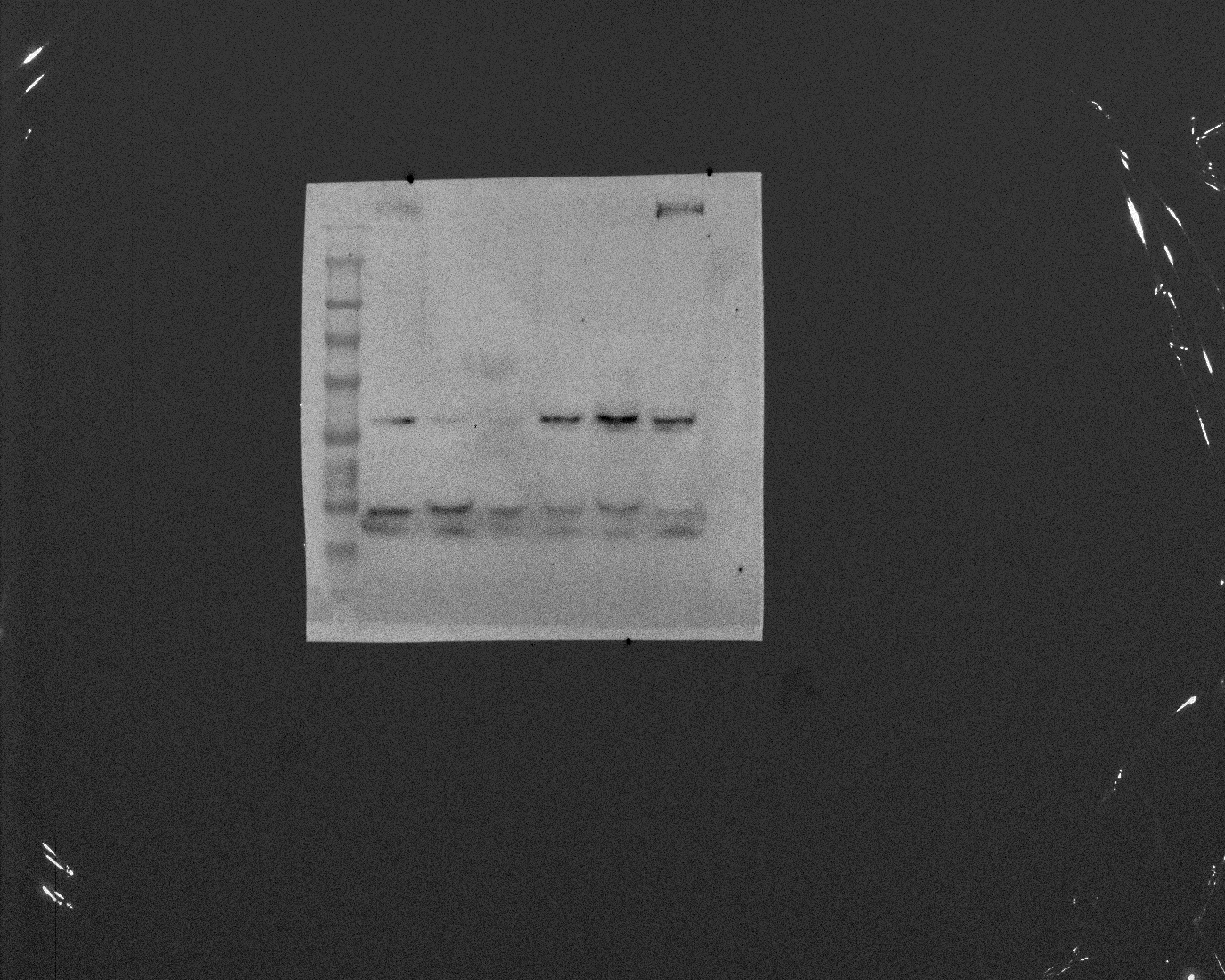

Supplement: Figure 6—figure supplement 1—source data 1. [file elife-81966-fig6-figsupp1-data1.zip › Figure 6-figure supplement 1-Source data 1.tif]

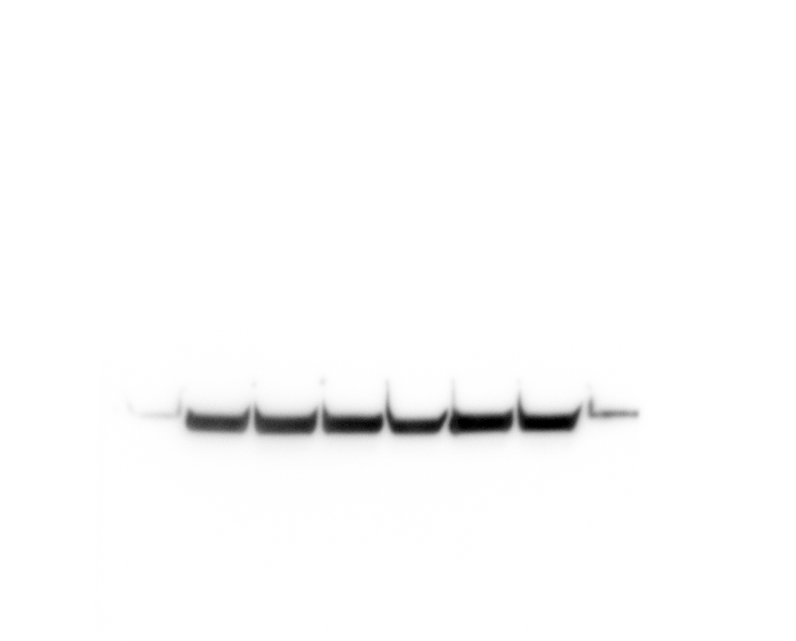

Supplement: Figure 6—figure supplement 1—source data 2. [file elife-81966-fig6-figsupp1-data2.zip › Figure 6-figure supplement 1-Source data 2.tif]

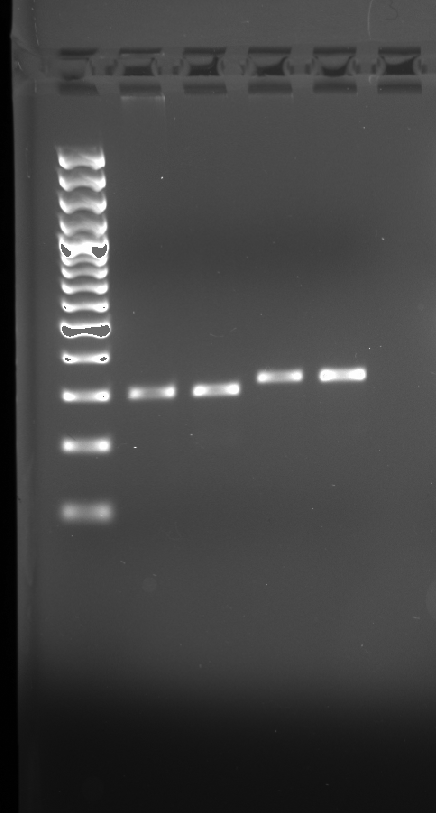

Supplement: Figure 6—figure supplement 1—source data 3. [file elife-81966-fig6-figsupp1-data3.zip › Figure 6-figure supplement 1-Source data 3.tif]

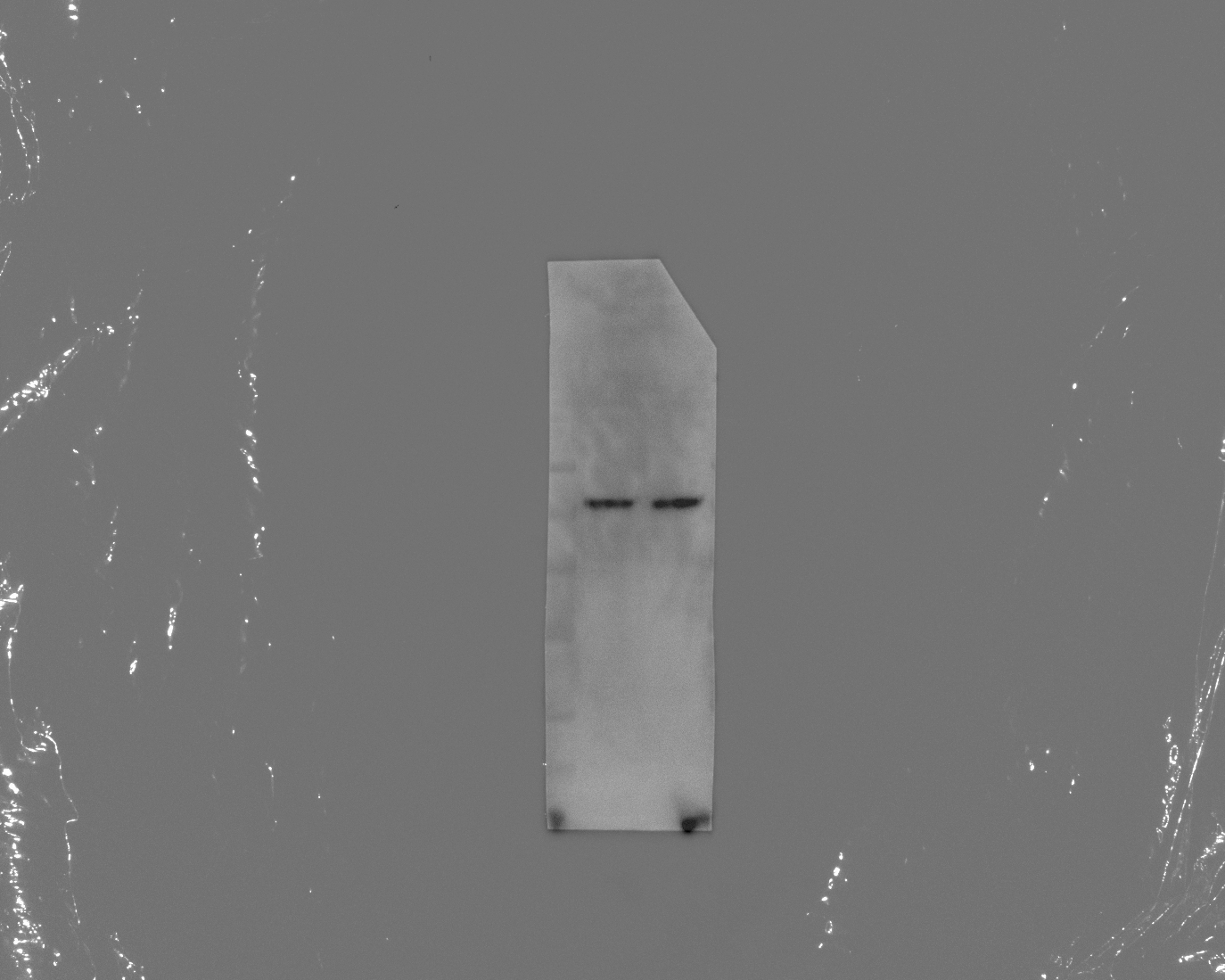

Supplement: Figure 6—figure supplement 1—source data 4. [file elife-81966-fig6-figsupp1-data4.zip › Figure 6-figure supplement 1-Source data 4 .tiff]

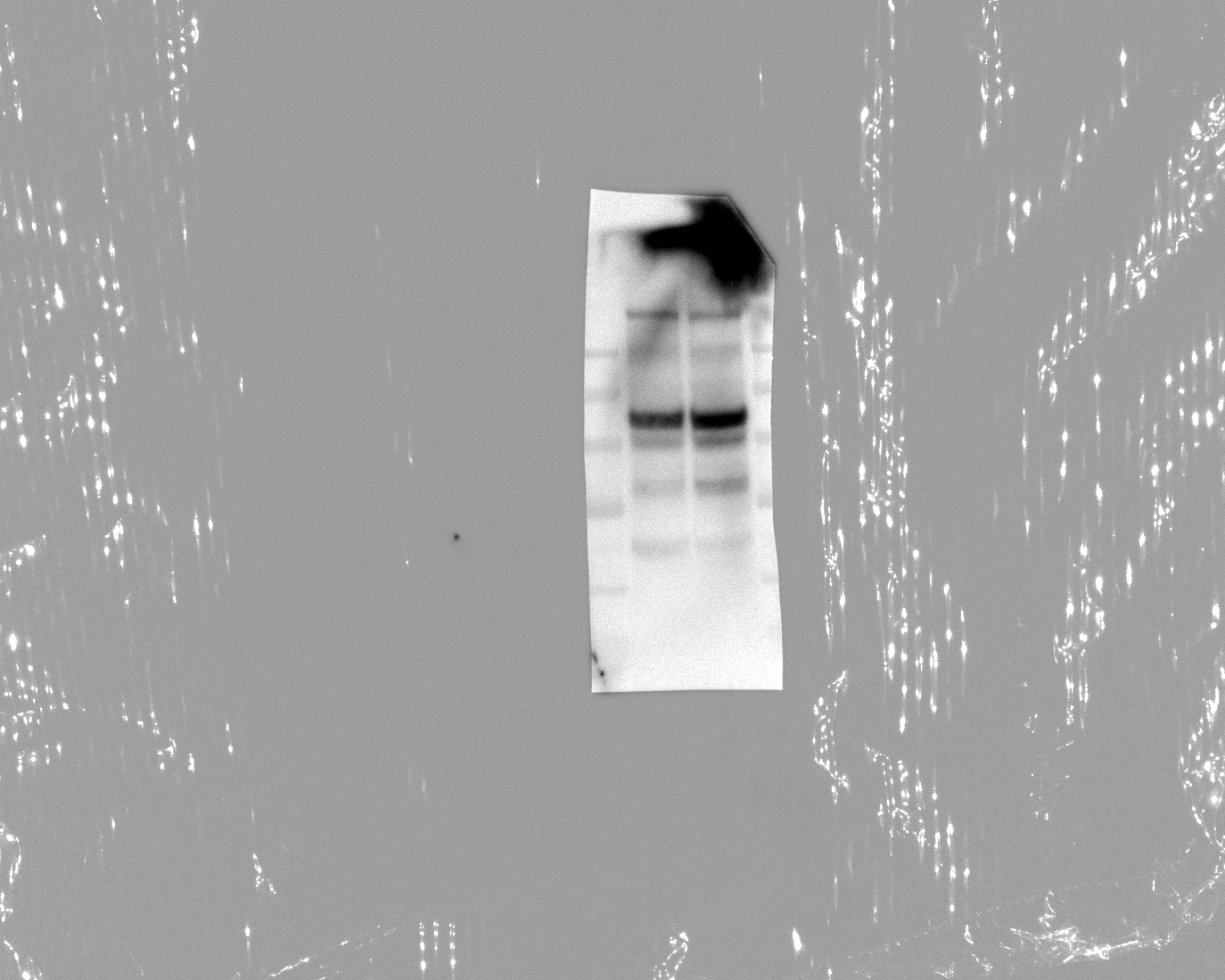

Supplement: Figure 6—figure supplement 1—source data 5. [file elife-81966-fig6-figsupp1-data5.zip › Figure 6-figure supplement 1-Source data 5.tif]

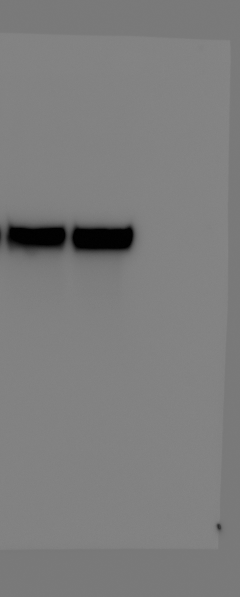

Supplement: Figure 6—figure supplement 1—source data 6. [file elife-81966-fig6-figsupp1-data6.zip › Figure 6-figure supplement 1-Source data 6.tif]

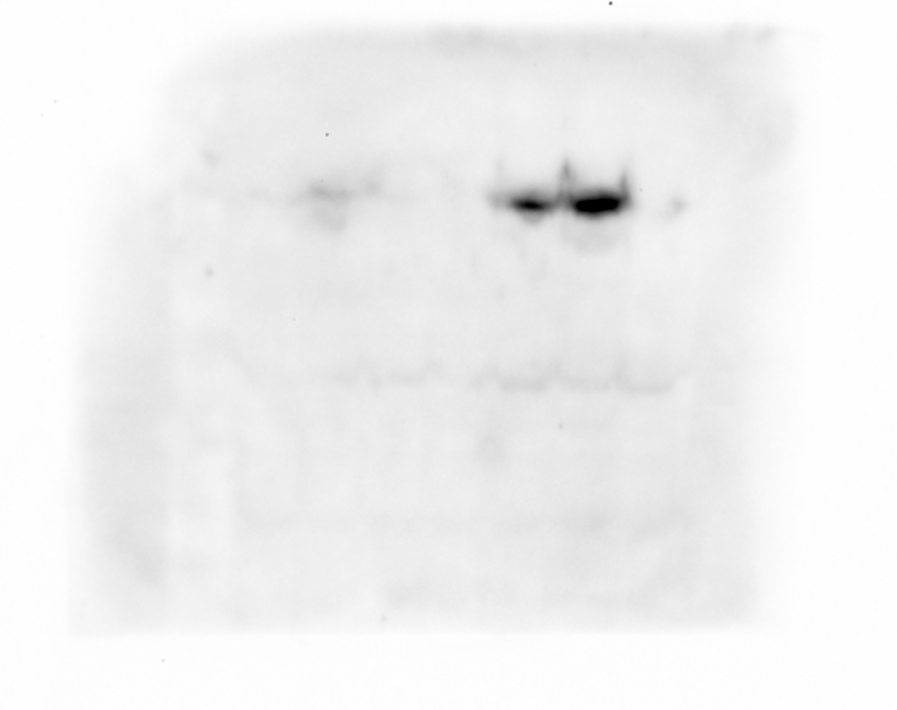

Supplement: Figure 7—source data 1. [file elife-81966-fig7-data1.zip › Figure 7 source data 1.tiff]

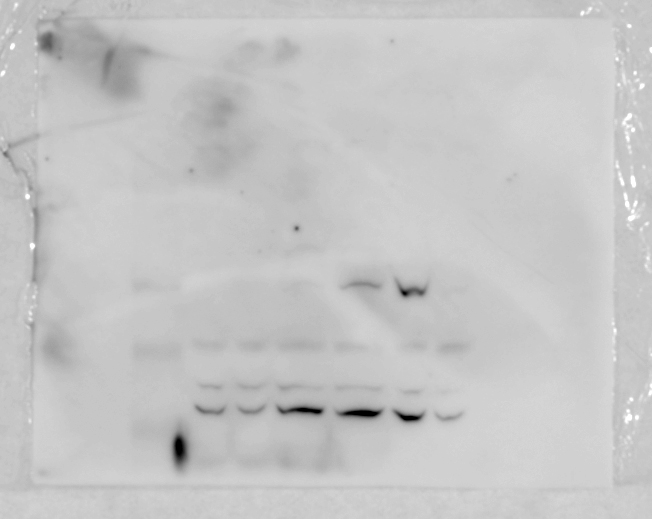

Supplement: Figure 7—source data 2. [file elife-81966-fig7-data2.zip › Figure 7 source data 2.tiff]

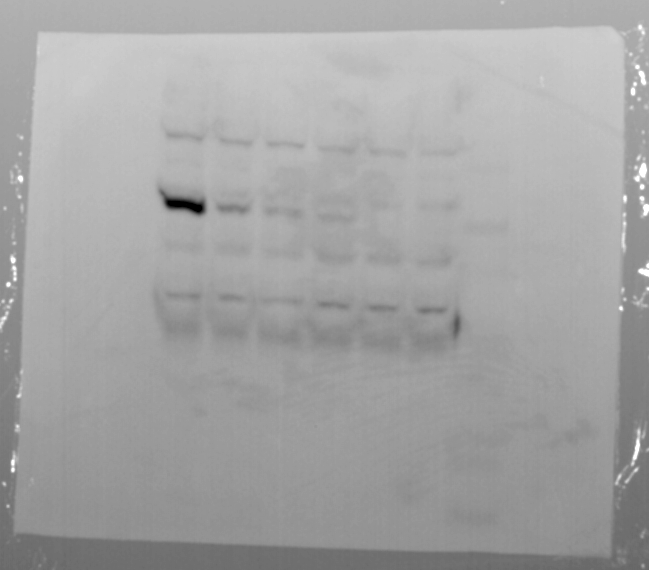

Supplement: Figure 7—source data 3. [file elife-81966-fig7-data3.zip › Figure 7 source data 3.tiff]

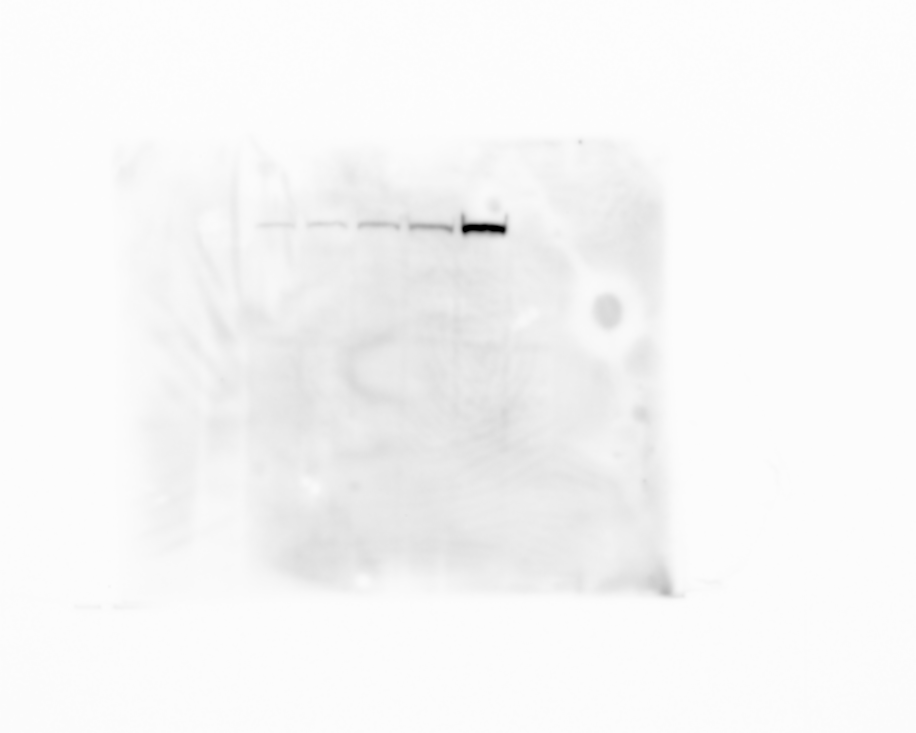

Supplement: Figure 7—source data 4. [file elife-81966-fig7-data4.zip › Figure 7 source data 4.tiff]

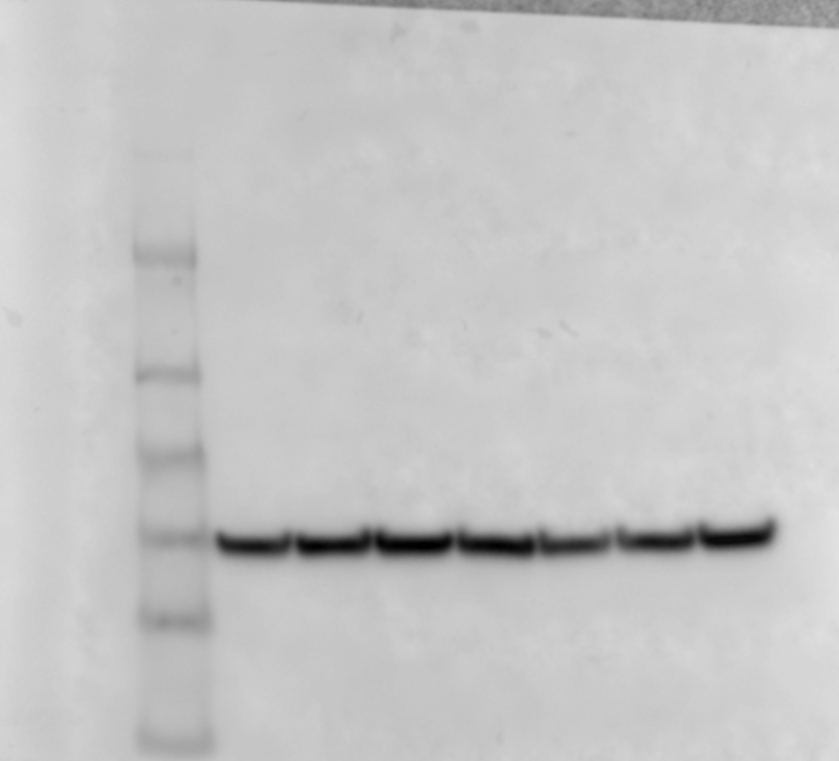

Supplement: Figure 7—source data 5. [file elife-81966-fig7-data5.zip › Figure 7 source data 5.tiff]

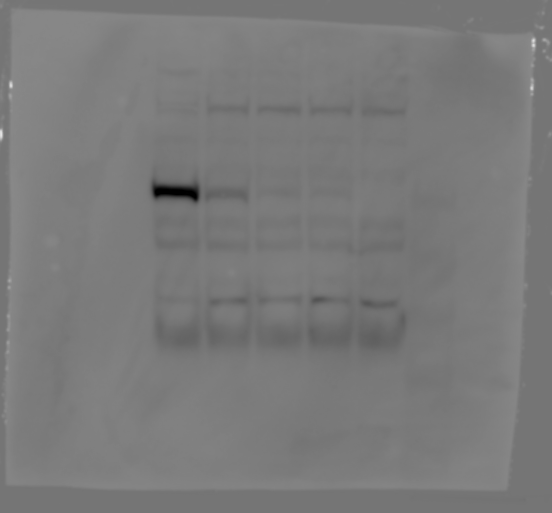

Supplement: Figure 7—source data 6. [file elife-81966-fig7-data6.zip › Figure 7 source data 6.tiff]

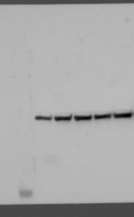

Supplement: Figure 7—source data 7. [file elife-81966-fig7-data7.zip › Figure 7 source data 7.tif]

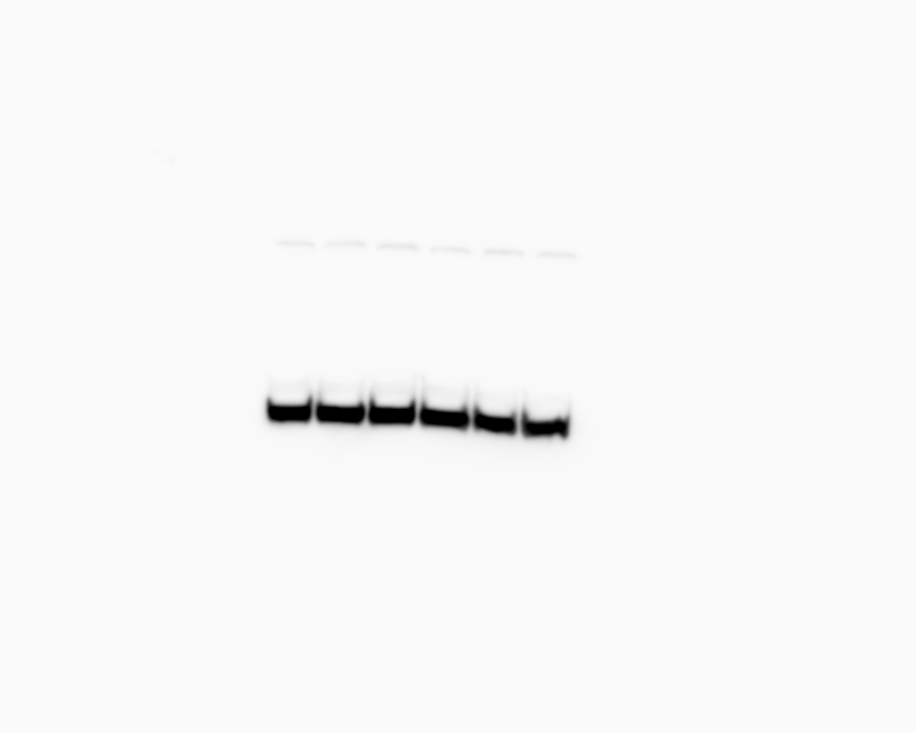

Supplement: Figure 7—source data 8. [file elife-81966-fig7-data8.zip › Figure 7 source data 8.tiff]

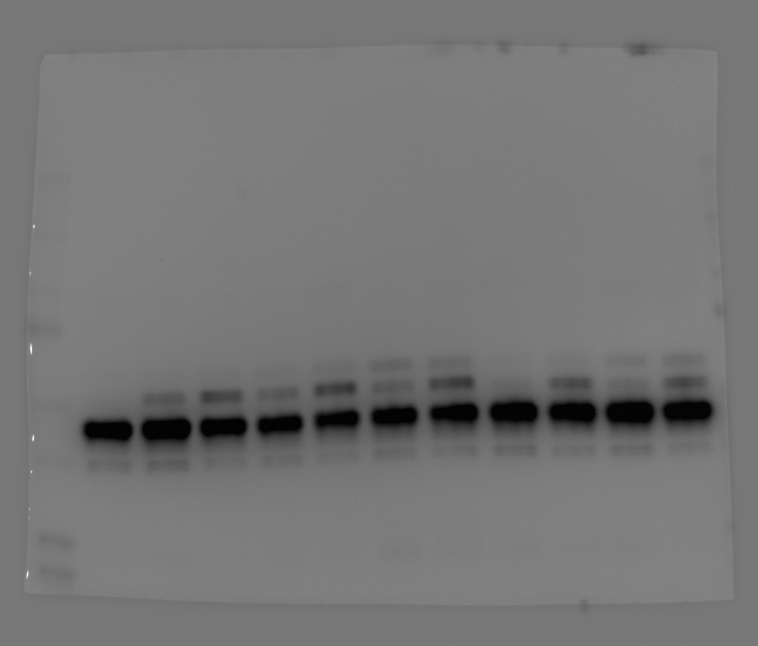

Supplement: Figure 7—source data 9. [file elife-81966-fig7-data9.zip › Figure 7 source data 9.tif]

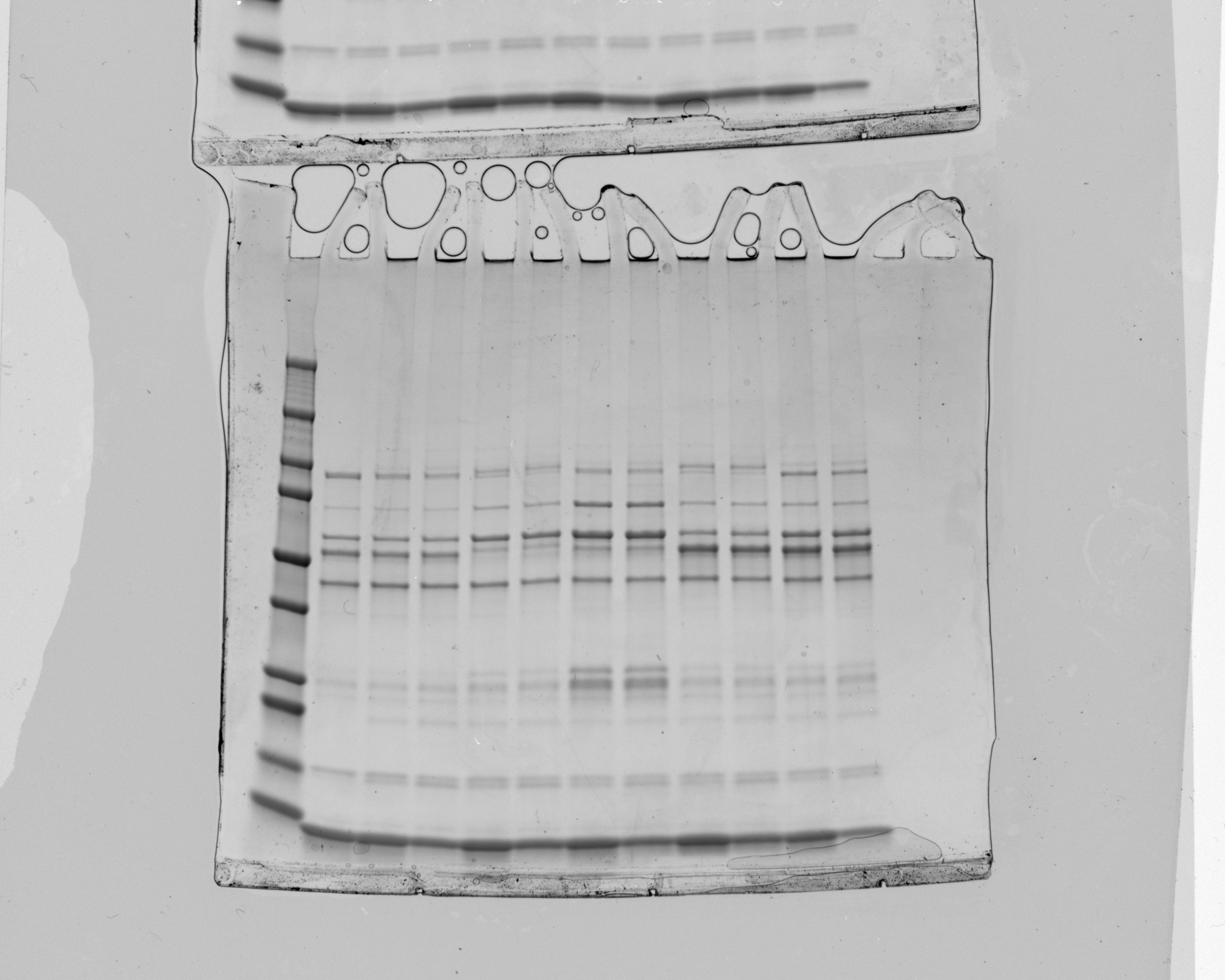

Supplement: Figure 7—source data 10. [file elife-81966-fig7-data10.zip › Figure 7 source data 10.tif]

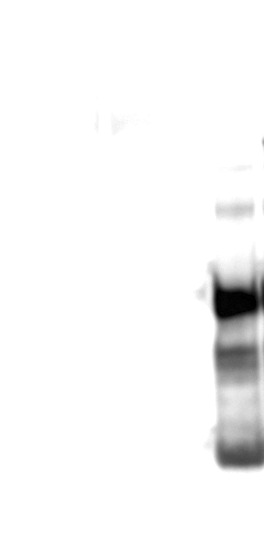

Supplement: Figure 7—source data 11. [file elife-81966-fig7-data11.zip › Figure 7 source data 11.tif]

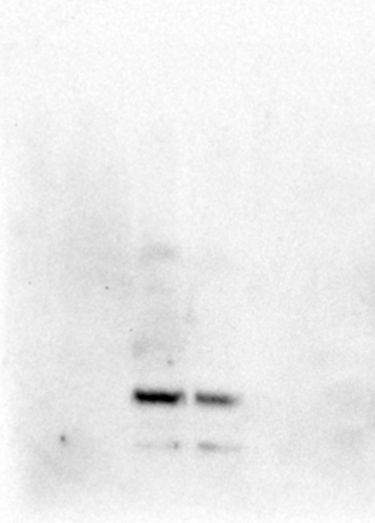

Supplement: Figure 7—source data 12. [file elife-81966-fig7-data12.zip › Figure 7 source data 12.tiff]

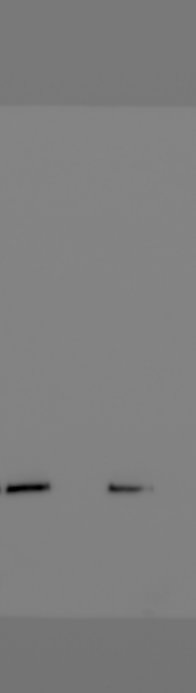

Supplement: Figure 7—figure supplement 1—source data 1. [file elife-81966-fig7-figsupp1-data1.zip › Figure 7-figure supplement 1-Source data 1.tif]

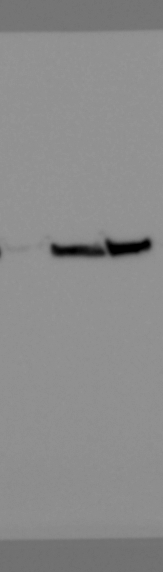

Supplement: Figure 7—figure supplement 1—source data 2. [file elife-81966-fig7-figsupp1-data2.zip › Figure 7-figure supplement 1-Source data 2.tif]

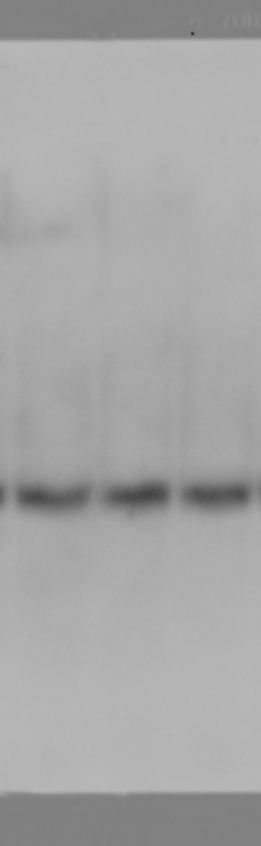

Supplement: Figure 7—figure supplement 1—source data 3. [file elife-81966-fig7-figsupp1-data3.zip › Figure 7-figure supplement 1-Source data 3.tif]

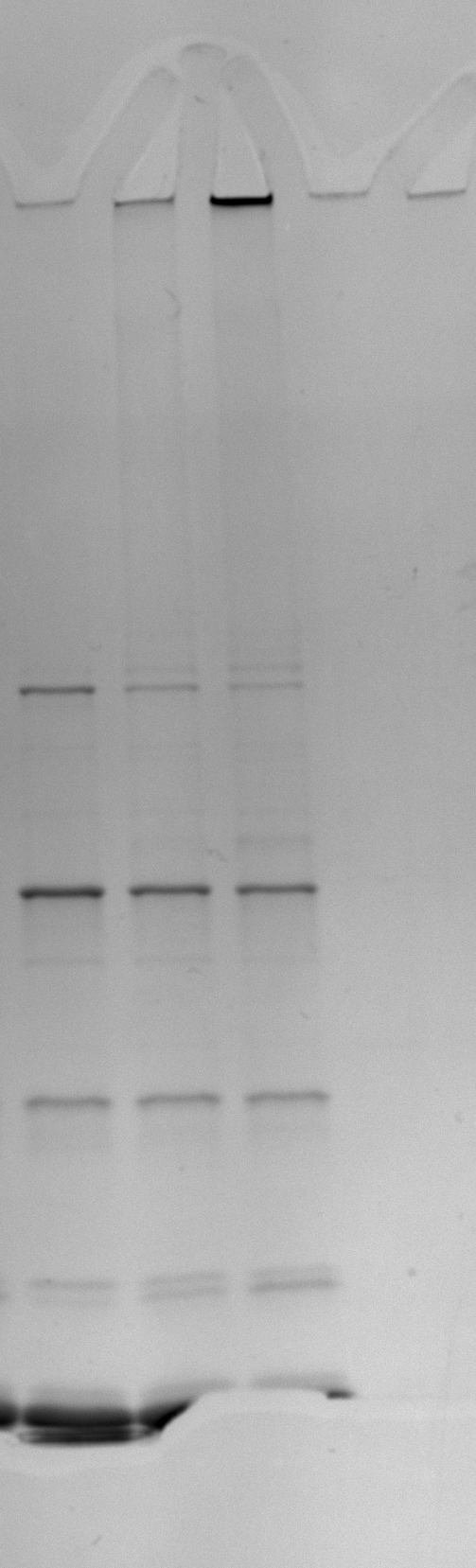

Supplement: Figure 7—figure supplement 1—source data 4. [file elife-81966-fig7-figsupp1-data4.zip › Figure 7-figure supplement 1-Source data 4.tif]

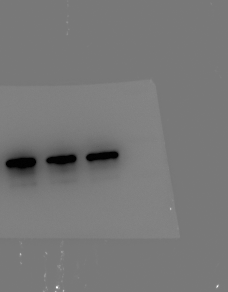

Supplement: Figure 7—figure supplement 1—source data 5. [file elife-81966-fig7-figsupp1-data5.zip › Figure 7-figure supplement 1-Source data 5.tif]

Figure 7-figure supplement 1-Source data 6

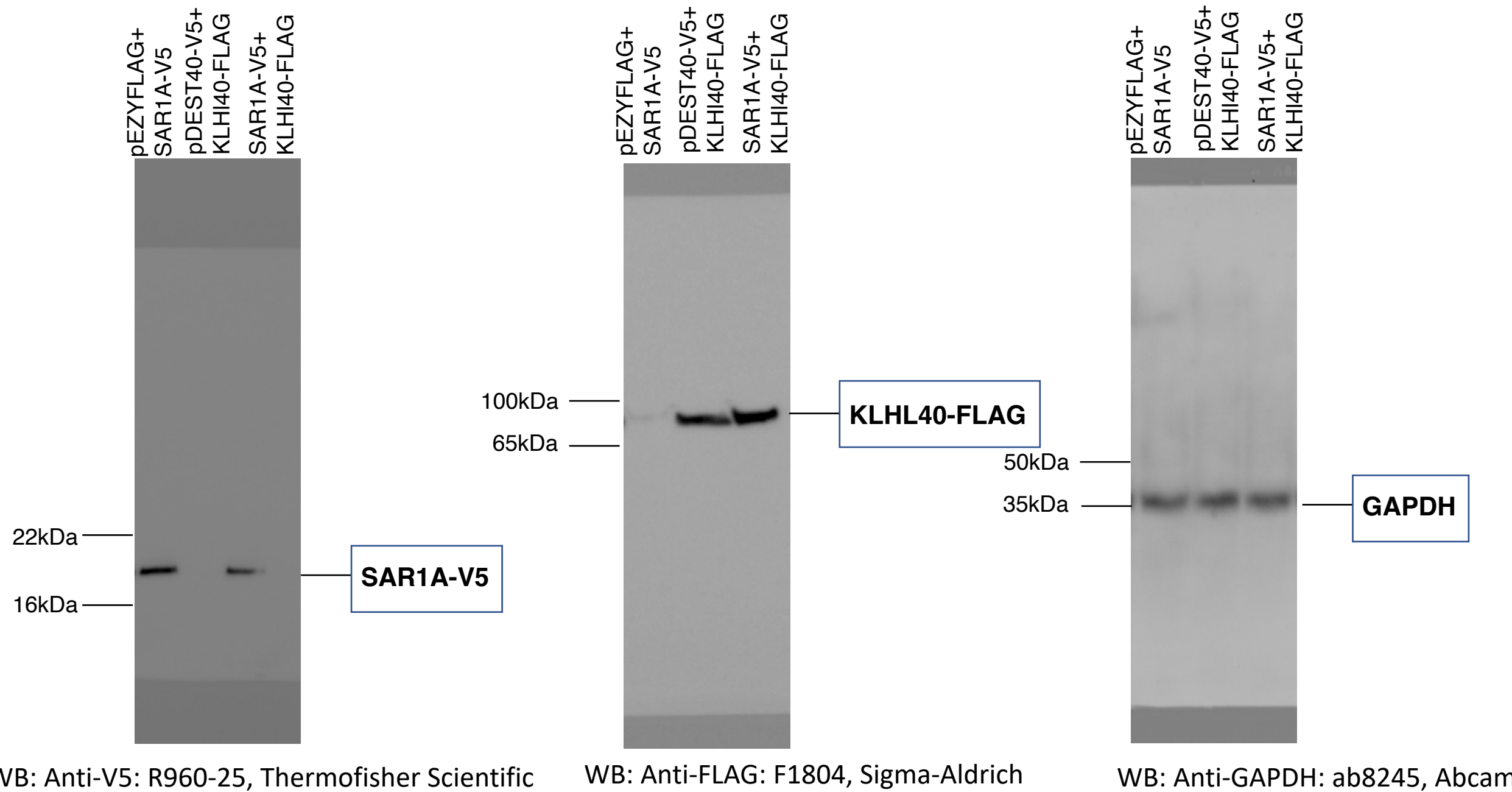

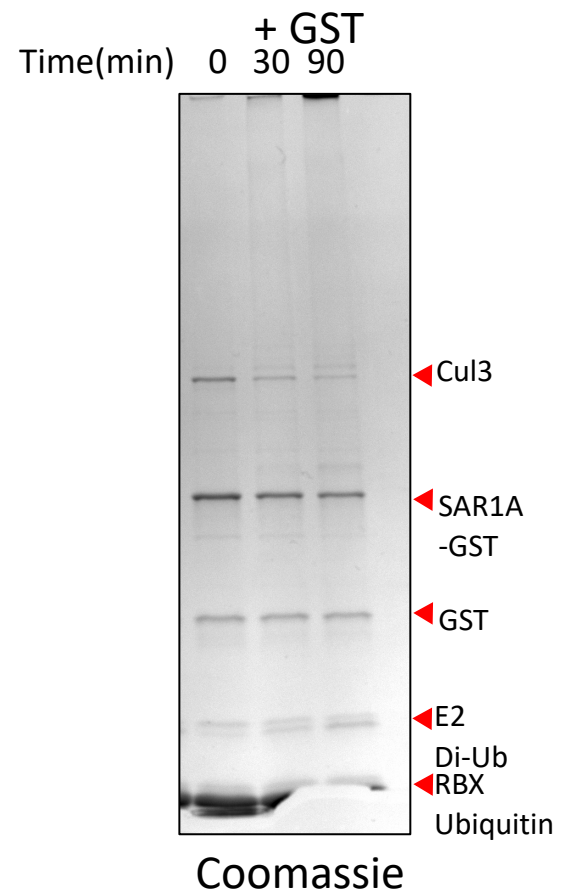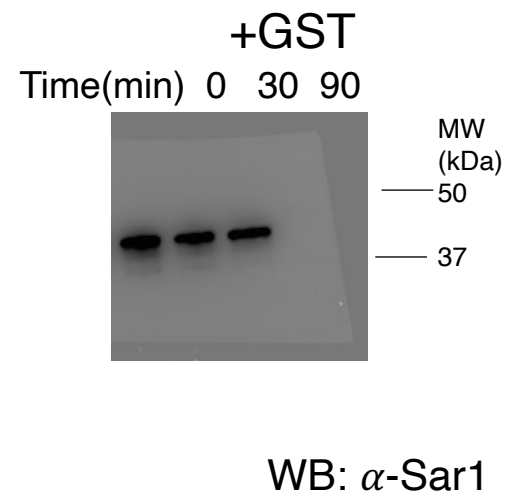

Supplement: Figure 7—figure supplement 1—source data 6. [file elife-81966-fig7-figsupp1-data6.pdf]

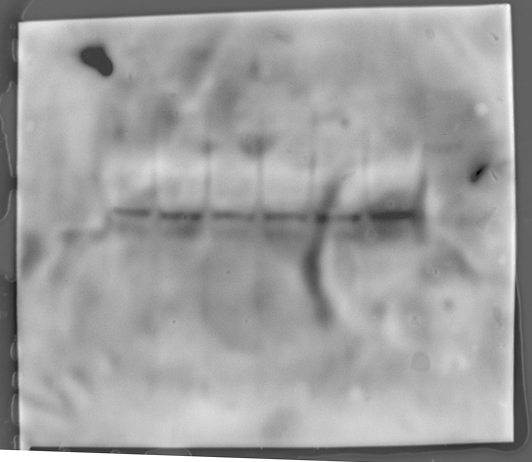

Supplement: Figure 9—figure supplement 1—source data 1. [file elife-81966-fig9-figsupp1-data1.zip › Figure 9-figure supplement 1-Source data 1.tiff]

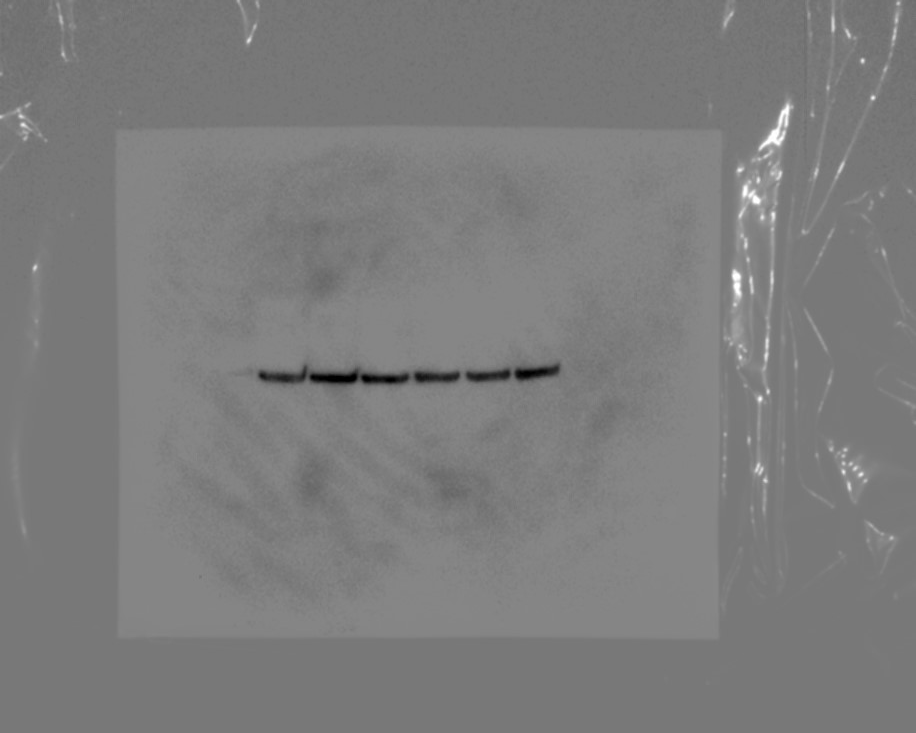

Supplement: Figure 9—figure supplement 1—source data 2. [file elife-81966-fig9-figsupp1-data2.zip › Figure 9-figure supplement 1-Source data 2.tiff]

Figure S9-figure supplement 1-Source data 3

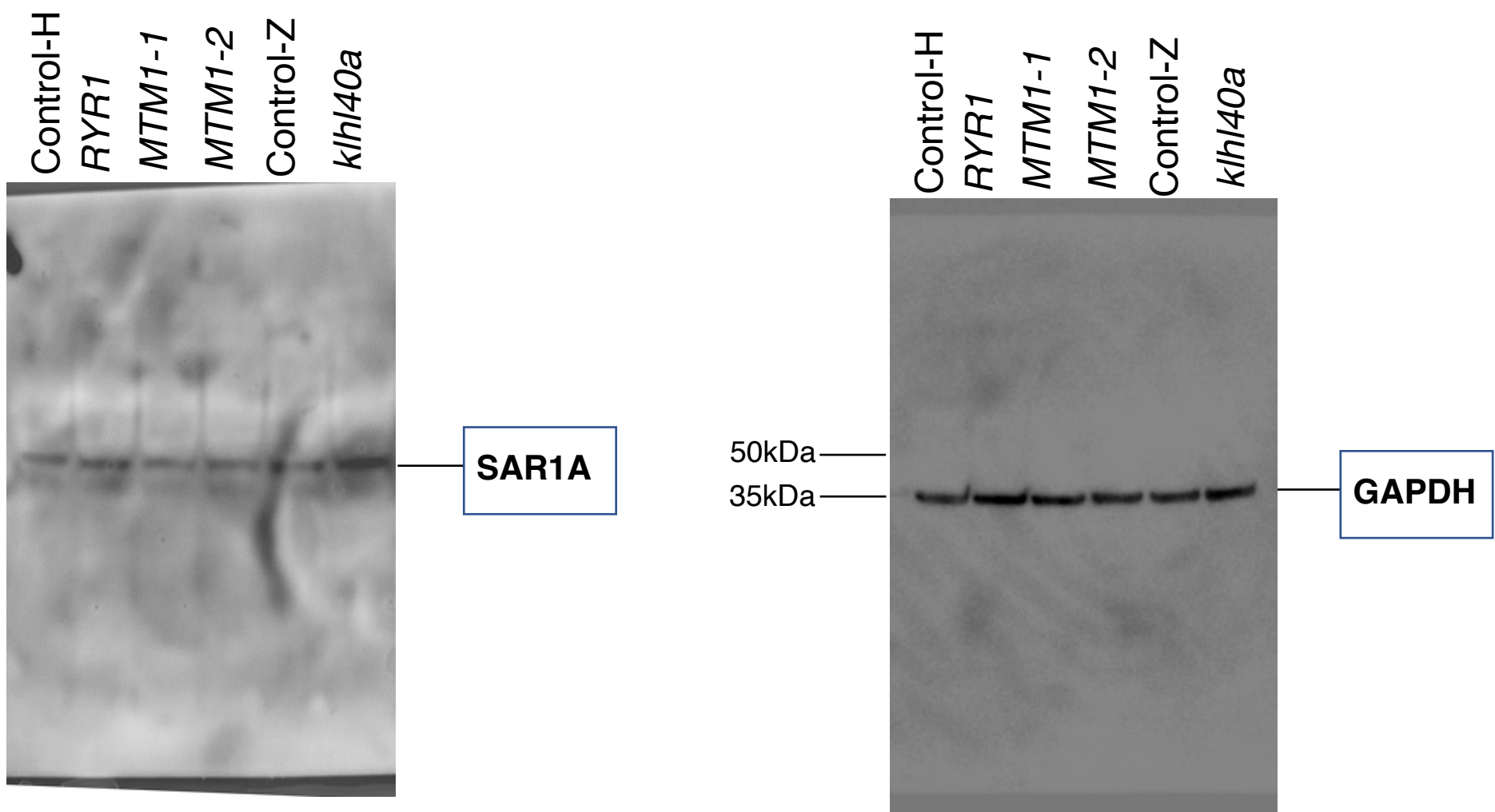

WB: Anti-Sar1: ab125871, Abcam

WB: Anti-GAPDH: ab8245, Abcam

Supplement: Figure 9—figure supplement 1—source data 3. [file elife-81966-fig9-figsupp1-data3.zip › Figure 9-figure supplement 1-Source data 3.pdf]
