# Supplementary material for: Dynamic regulation of inter-organelle communication by ubiquitylation controls skeletal muscle development and disease onset
Source: eLife. 2023 Jul 11;12:e81966. doi: 10.7554/eLife.81966 (PMC10356137; doi:10.7554/eLife.81966)
Supplement: Figure 1—figure supplement 1—source data 4. [file elife-81966-fig1-figsupp1-data4.pdf]

Figure 1-figure supplement 1-source data 4

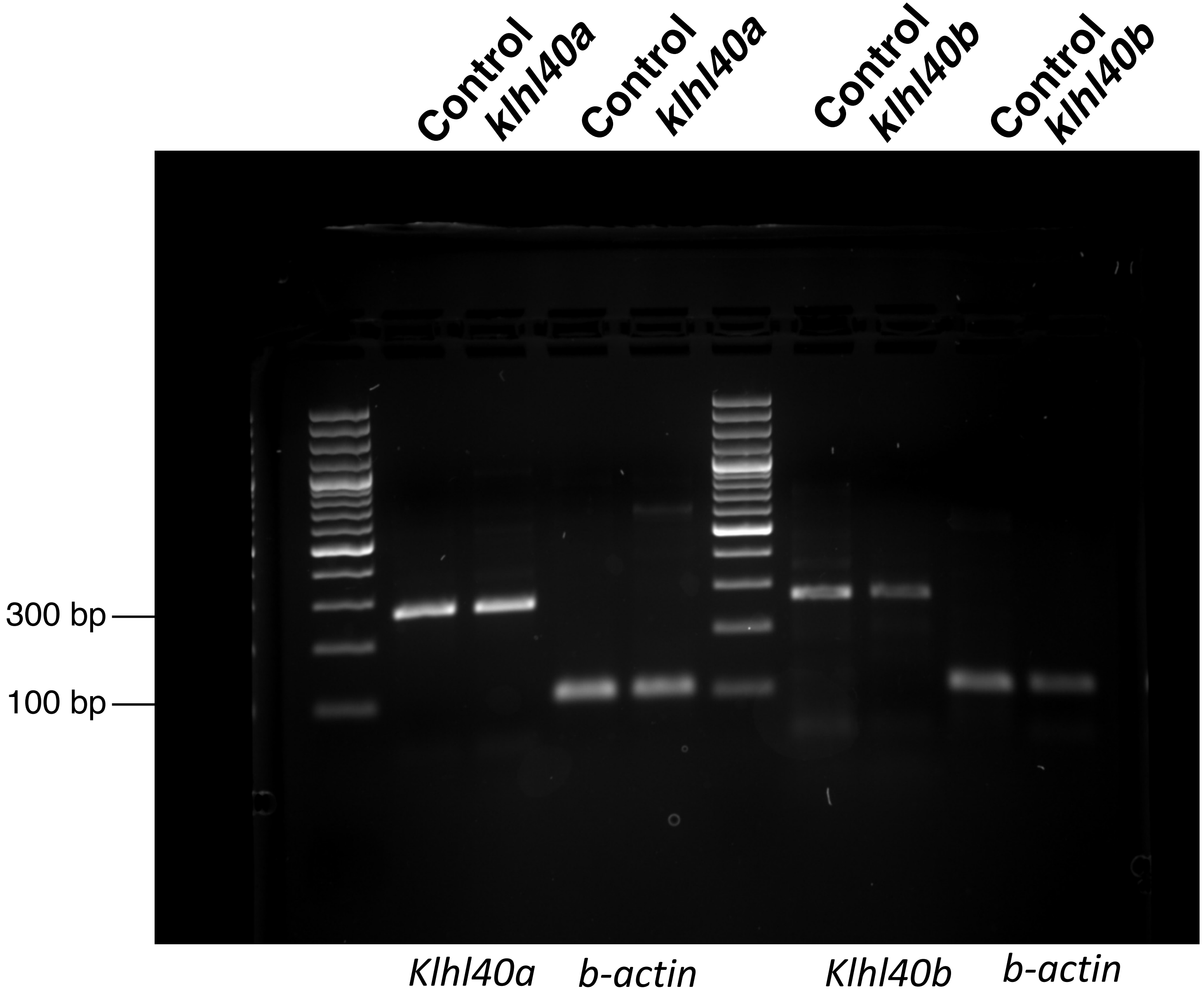

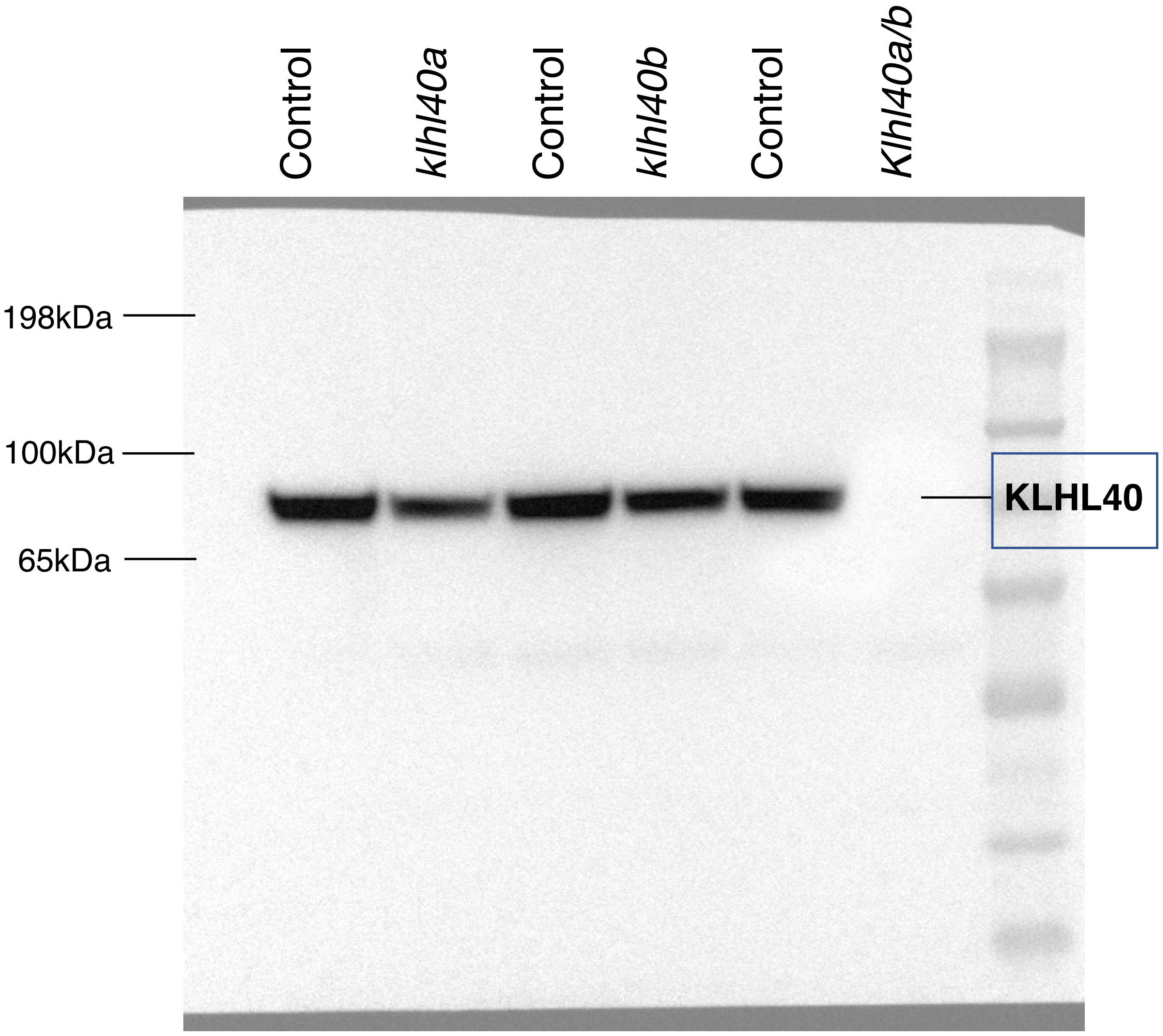

WB: Anti-KBTBD5: sc-99943, Santa Cruz Biotechnology

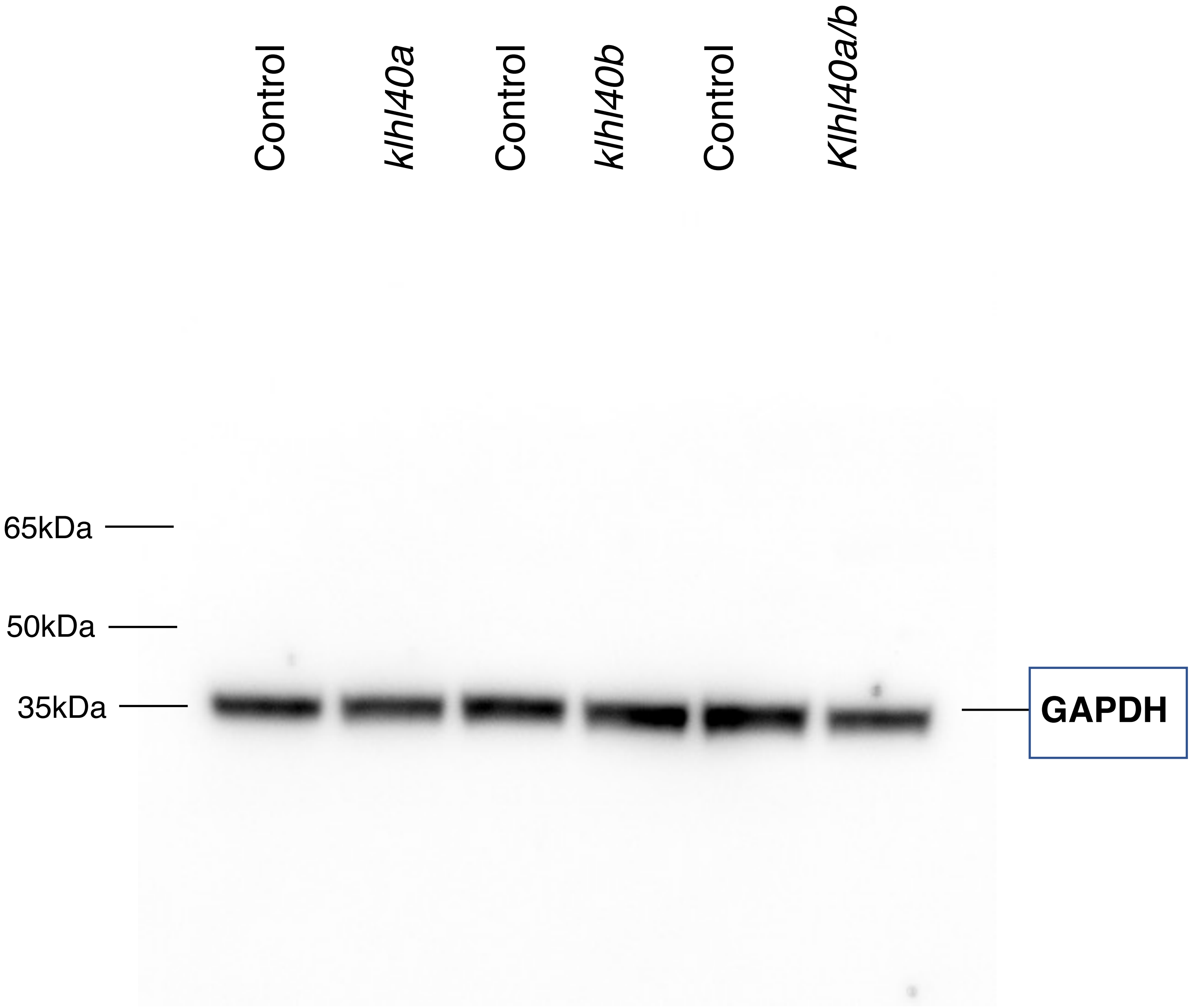

WB: Anti-GAPDH: 2118, Cell Signaling Technology
