# Supplementary material for: Dynamic regulation of inter-organelle communication by ubiquitylation controls skeletal muscle development and disease onset
Source: eLife. 2023 Jul 11;12:e81966. doi: 10.7554/eLife.81966 (PMC10356137; doi:10.7554/eLife.81966)
Supplement: Figure 5—source data 5. [file elife-81966-fig5-data5.pdf]

Figure 5 source file 5

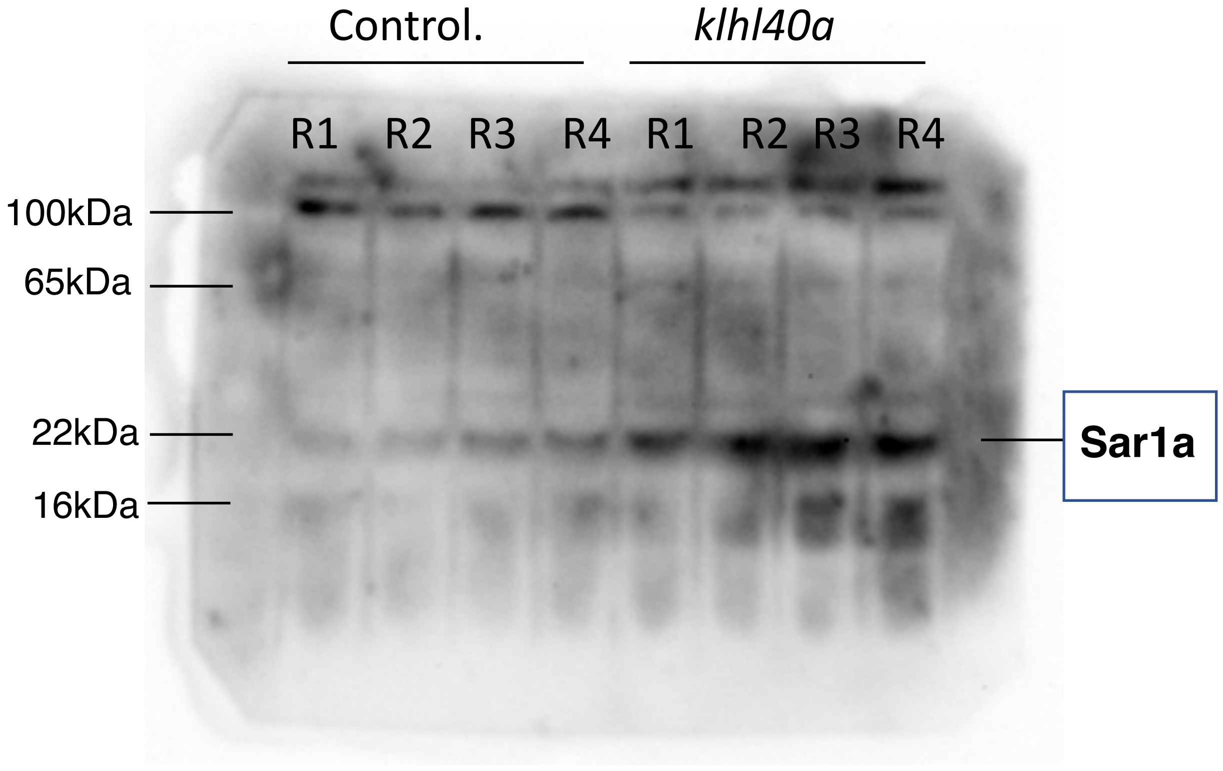

WB: Anti-Sar1: ab125871, Abcam

Biological Replicates

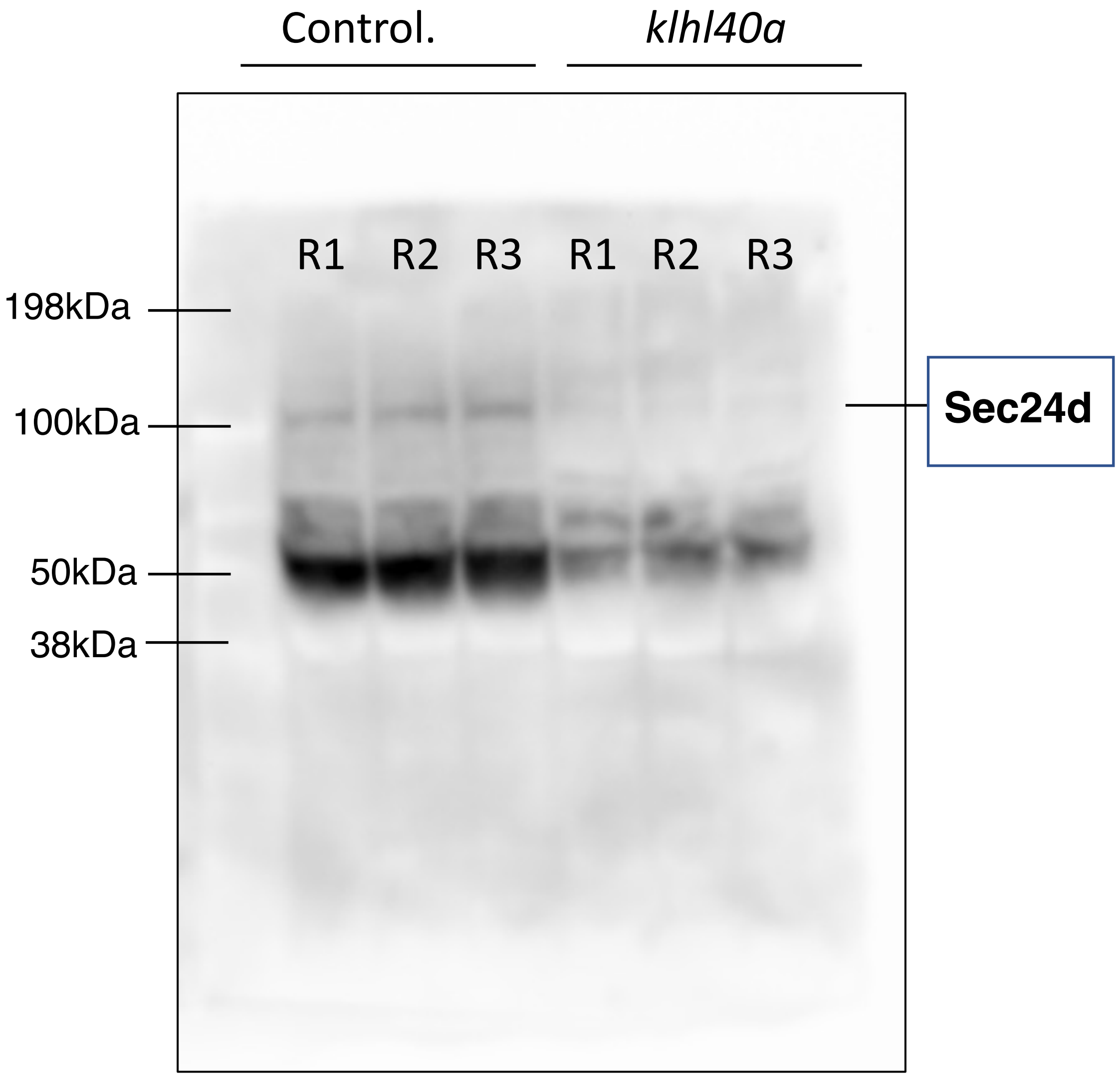

WB: Anti-Sec24d: 14687, Cell Signaling Technology

Figure 5 source file 5

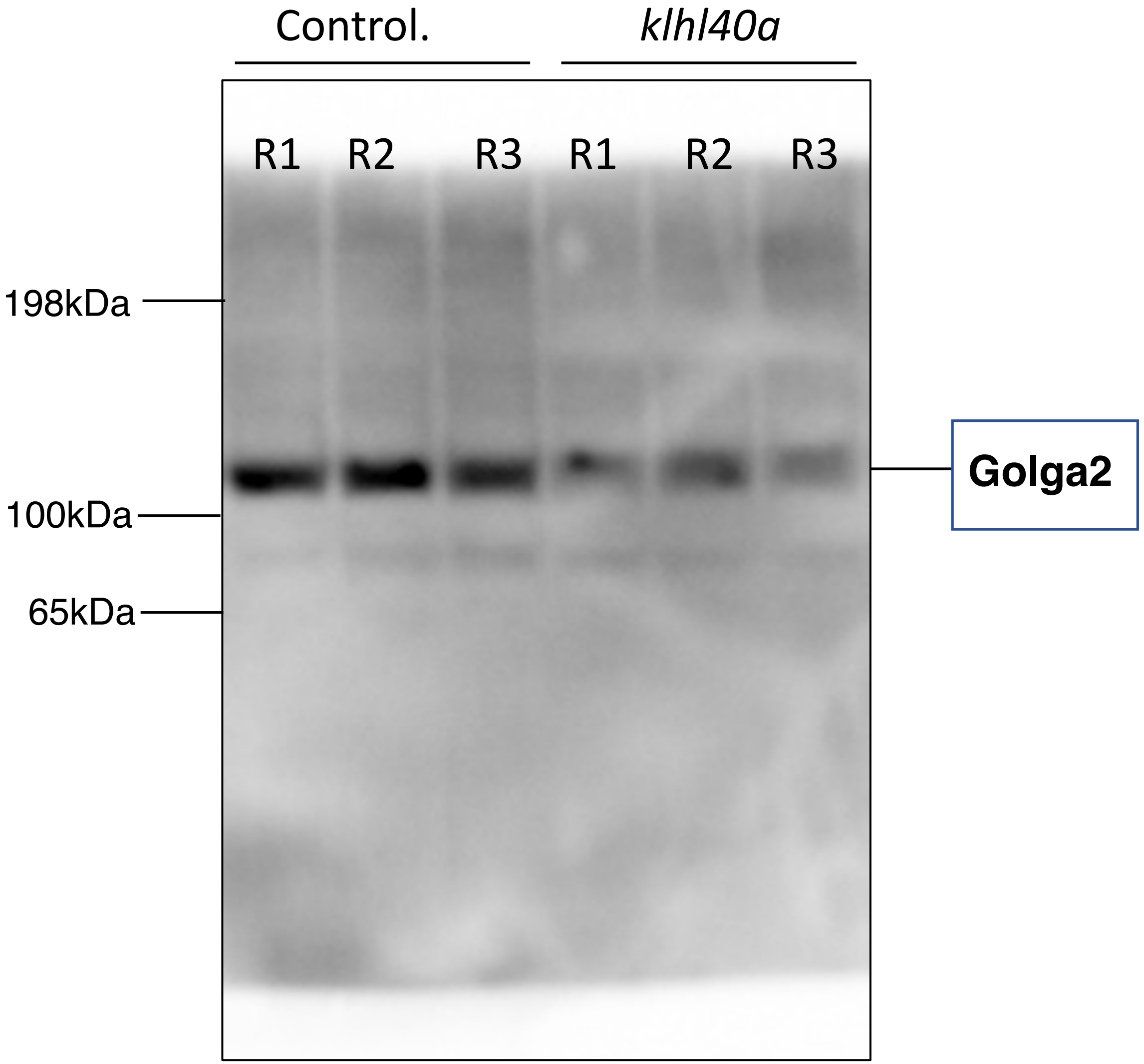

WB: Anti-Golga2: ab30637, Abcam

Biological Replicates

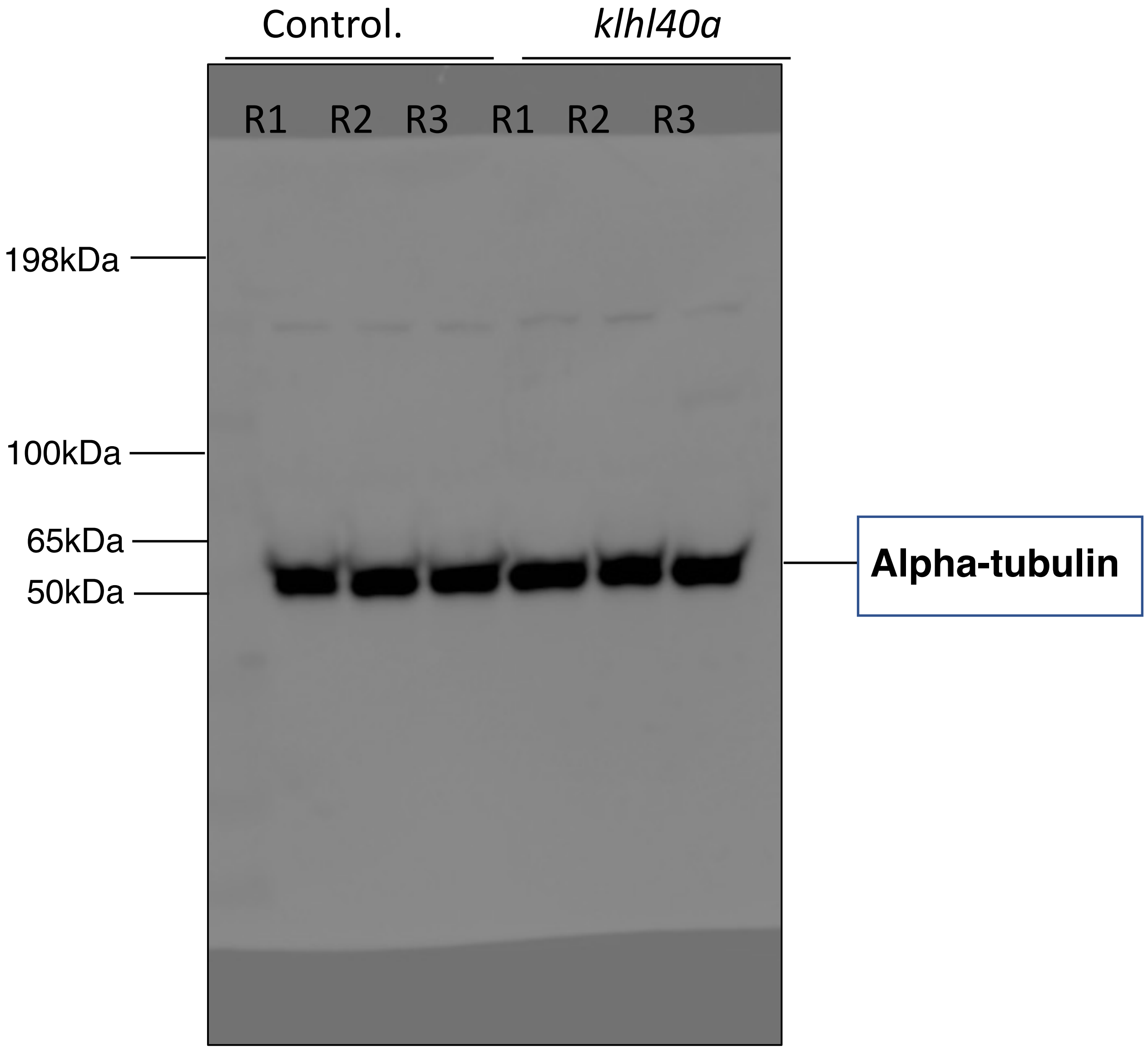

WB: Anti-tubulin: ab18251, Abcam
