# Supplementary material for: Dynamic regulation of inter-organelle communication by ubiquitylation controls skeletal muscle development and disease onset
Source: eLife. 2023 Jul 11;12:e81966. doi: 10.7554/eLife.81966 (PMC10356137; doi:10.7554/eLife.81966)
Supplement: Figure 6—figure supplement 1—source data 7. [file elife-81966-fig6-figsupp1-data7.pdf]

Figure 6-figure supplement 1-Source data 7

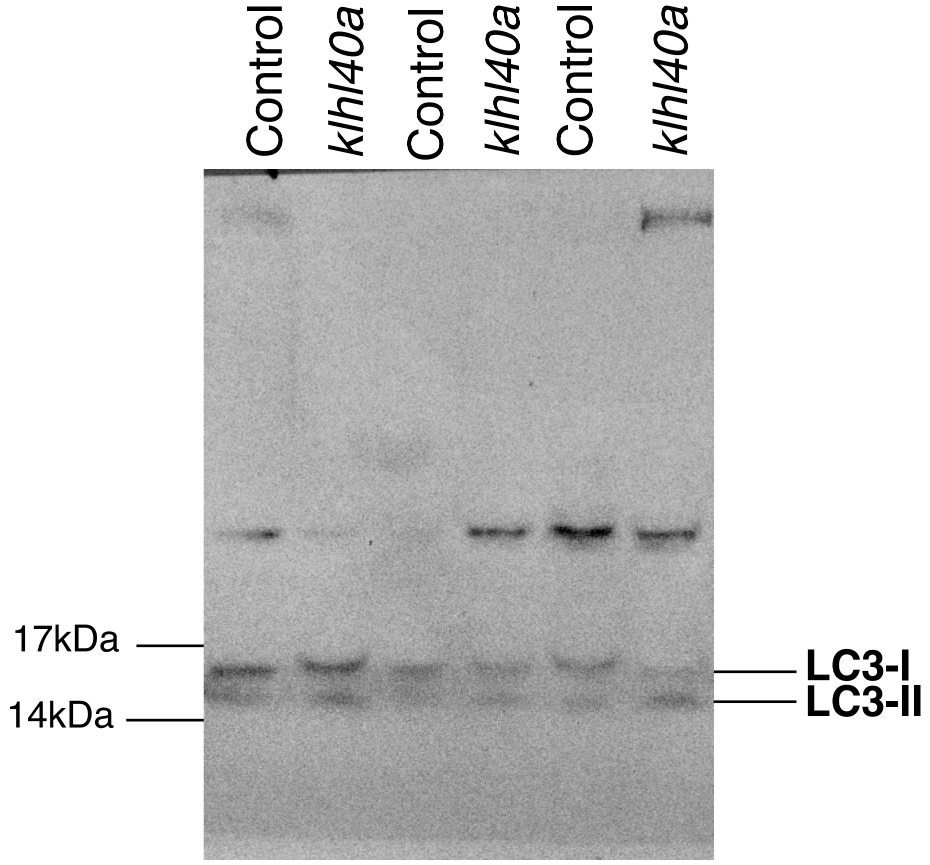

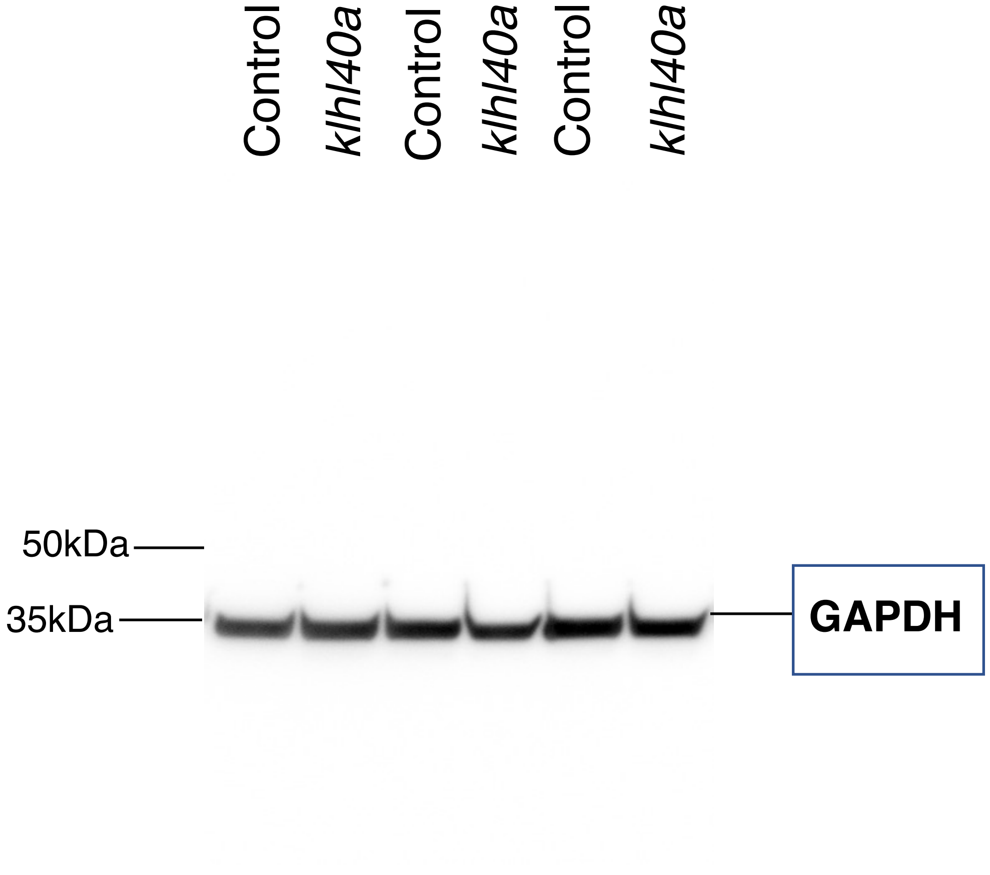

WB: Anti-LC3: 3868, Cell Signaling

WB: Anti-GAPDH: ab8245, Abcam

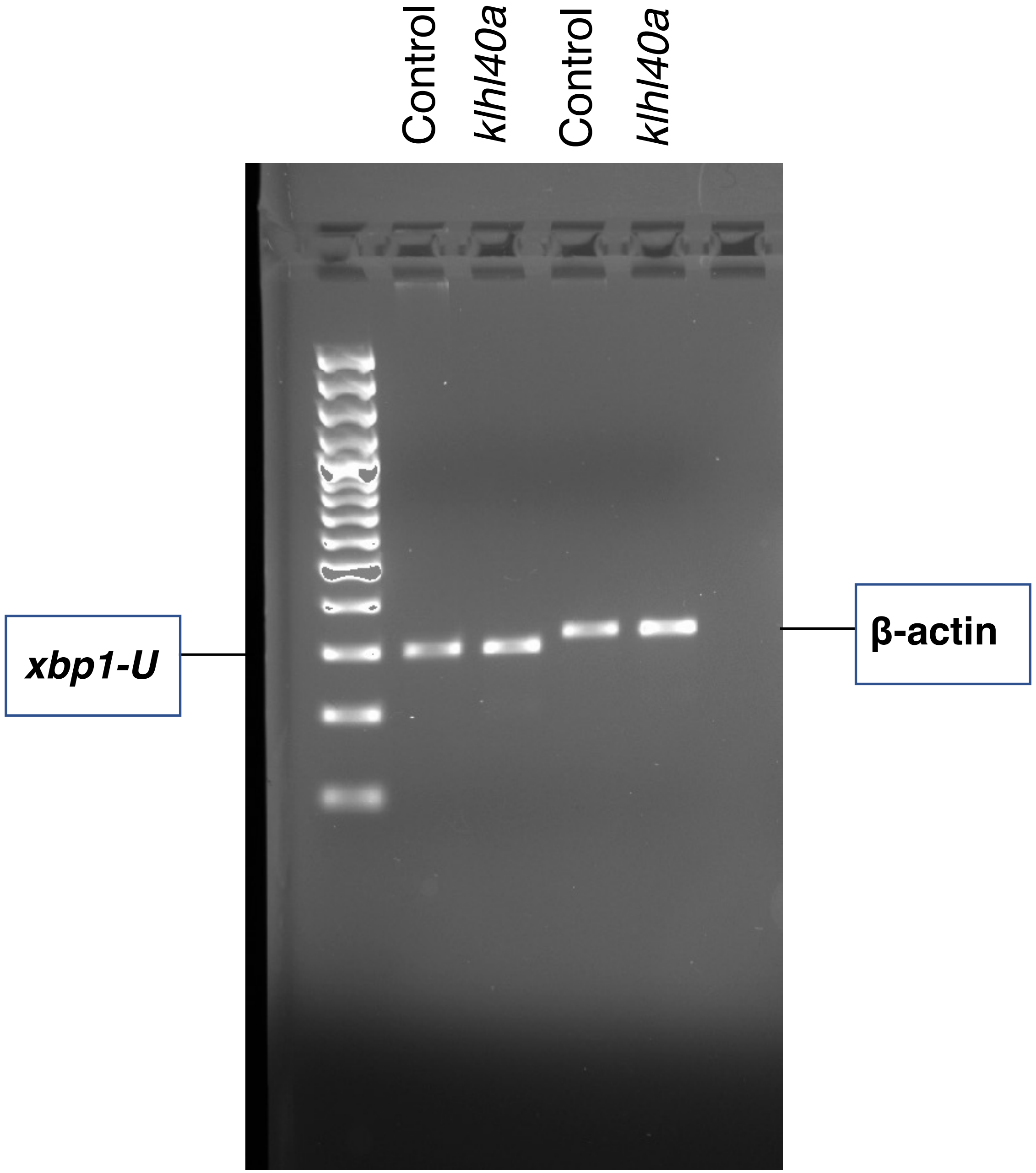
Control
klhl40a
Control
klhl40a
xbp1-U
β-actin

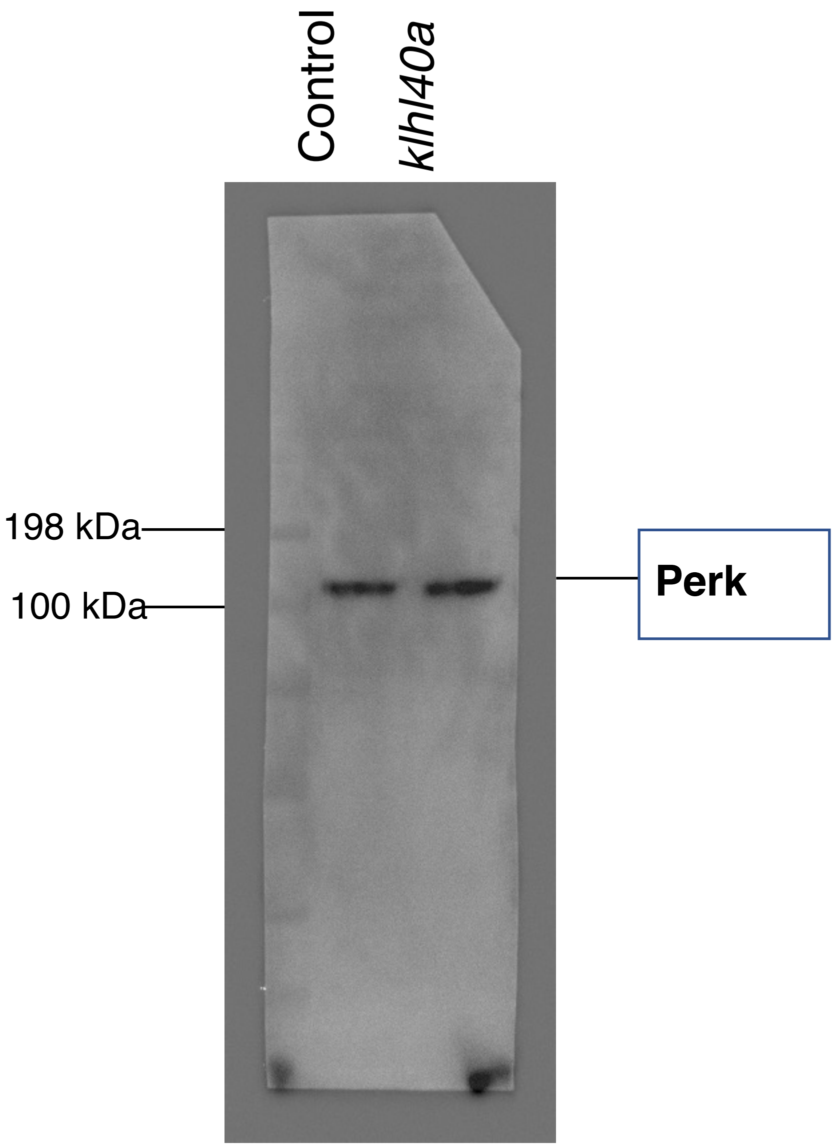

WB: Anti-PERK (3192, Cell Signaling Technology)

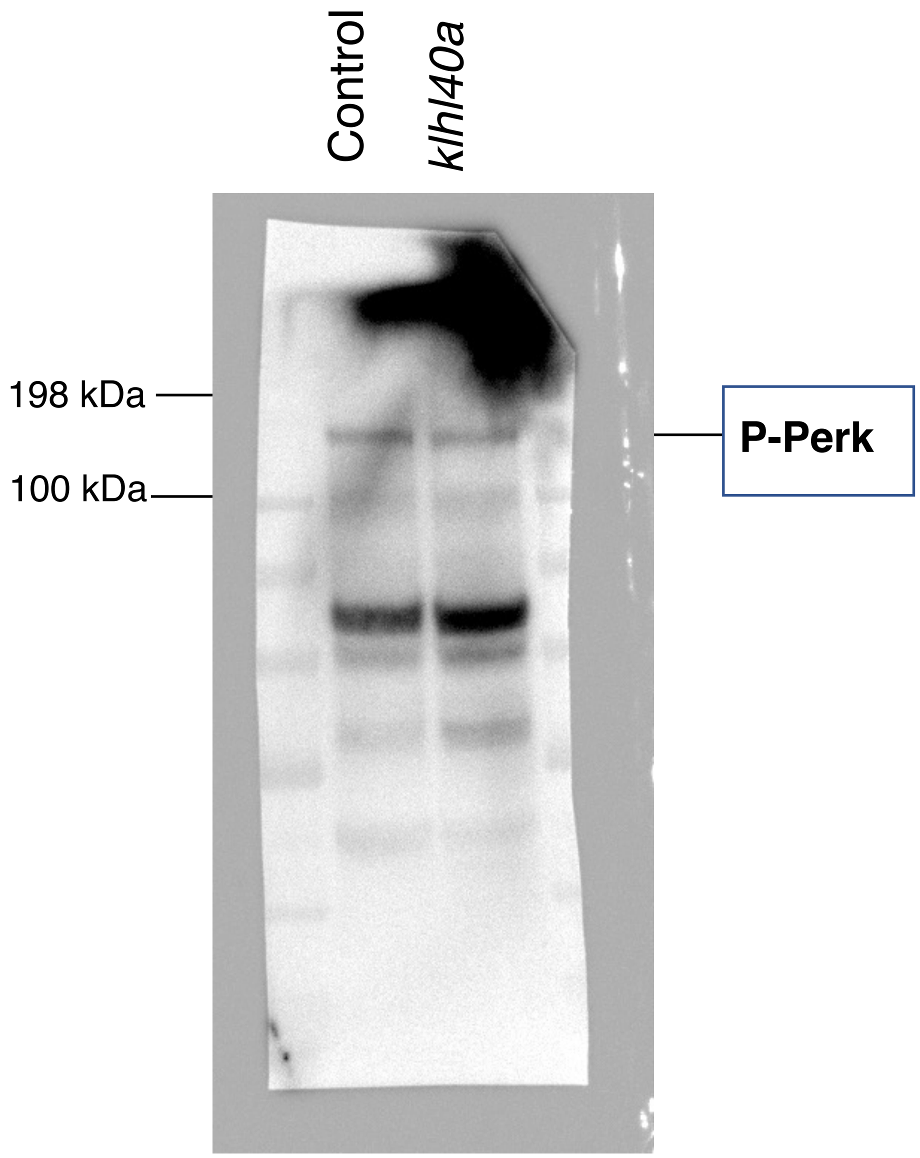

WB: Anti P-perk (3179, Cell Signaling Technology)

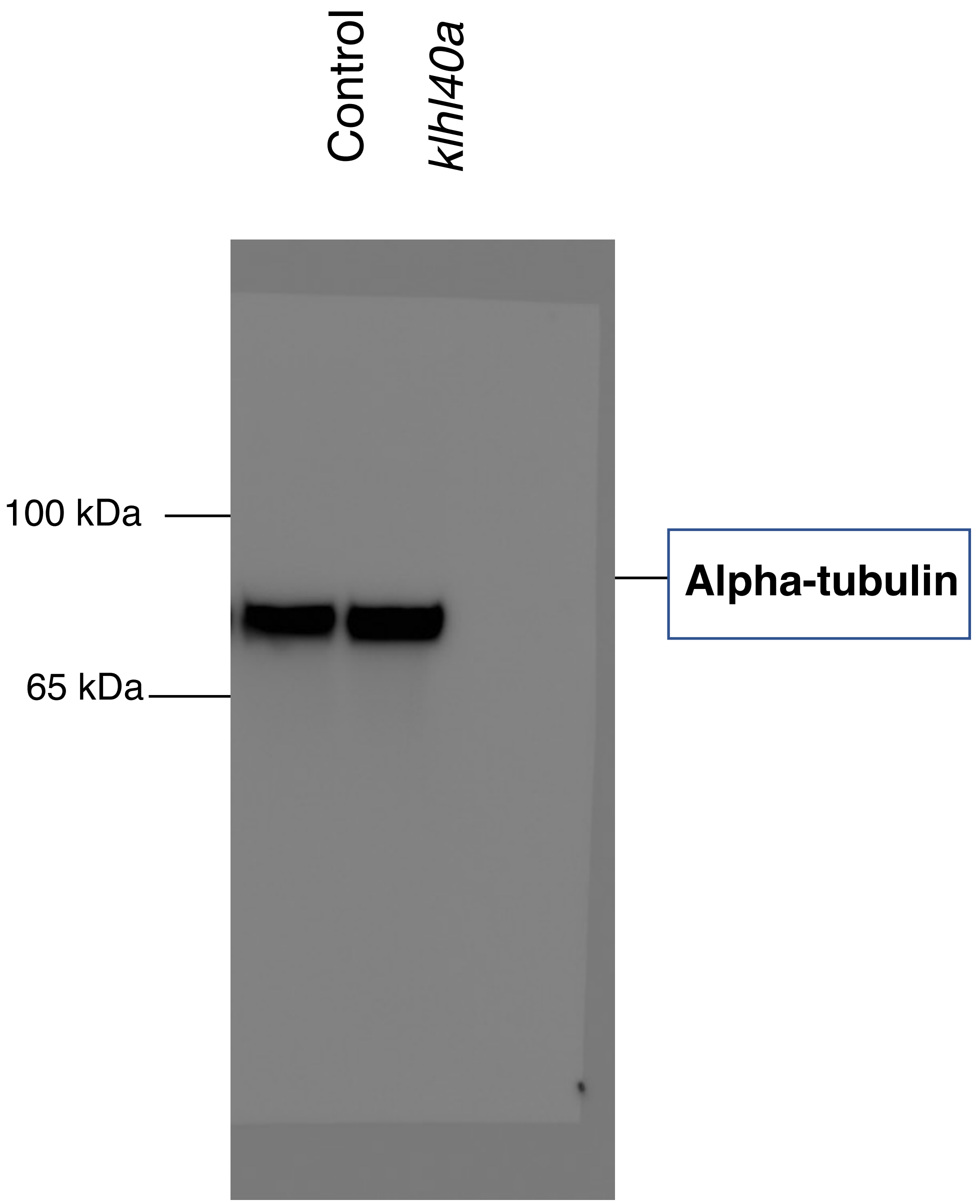

WB: Anti-alpha tubulin (ab18251, Abcam)
