# Supplementary material for: Dynamic regulation of inter-organelle communication by ubiquitylation controls skeletal muscle development and disease onset
Source: eLife. 2023 Jul 11;12:e81966. doi: 10.7554/eLife.81966 (PMC10356137; doi:10.7554/eLife.81966)
Supplement: Figure 7—source data 13. [file elife-81966-fig7-data13.pdf]

Figure 7A

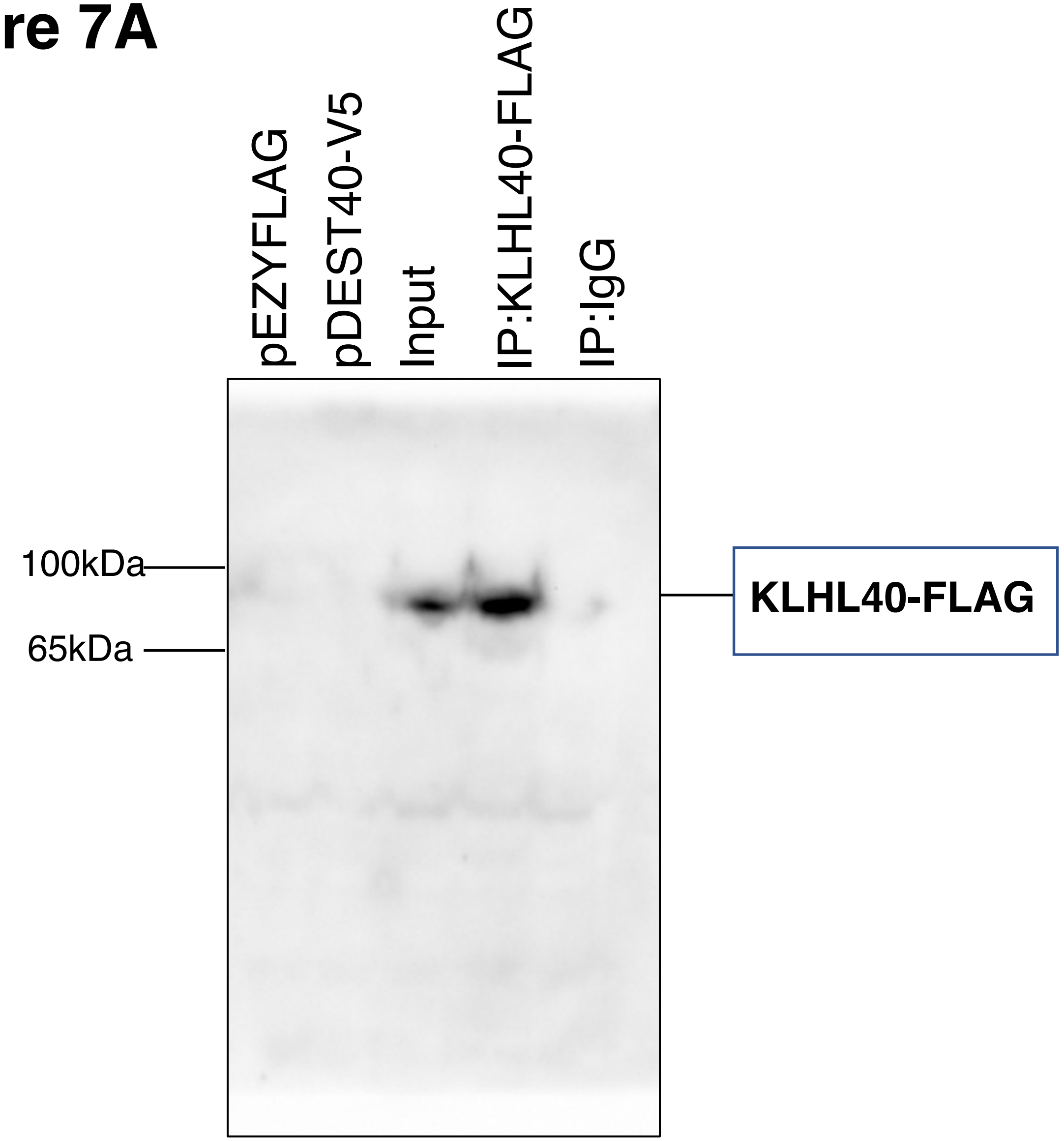

WB: Anti-FLAG: F1804, Sigma-Aldrich

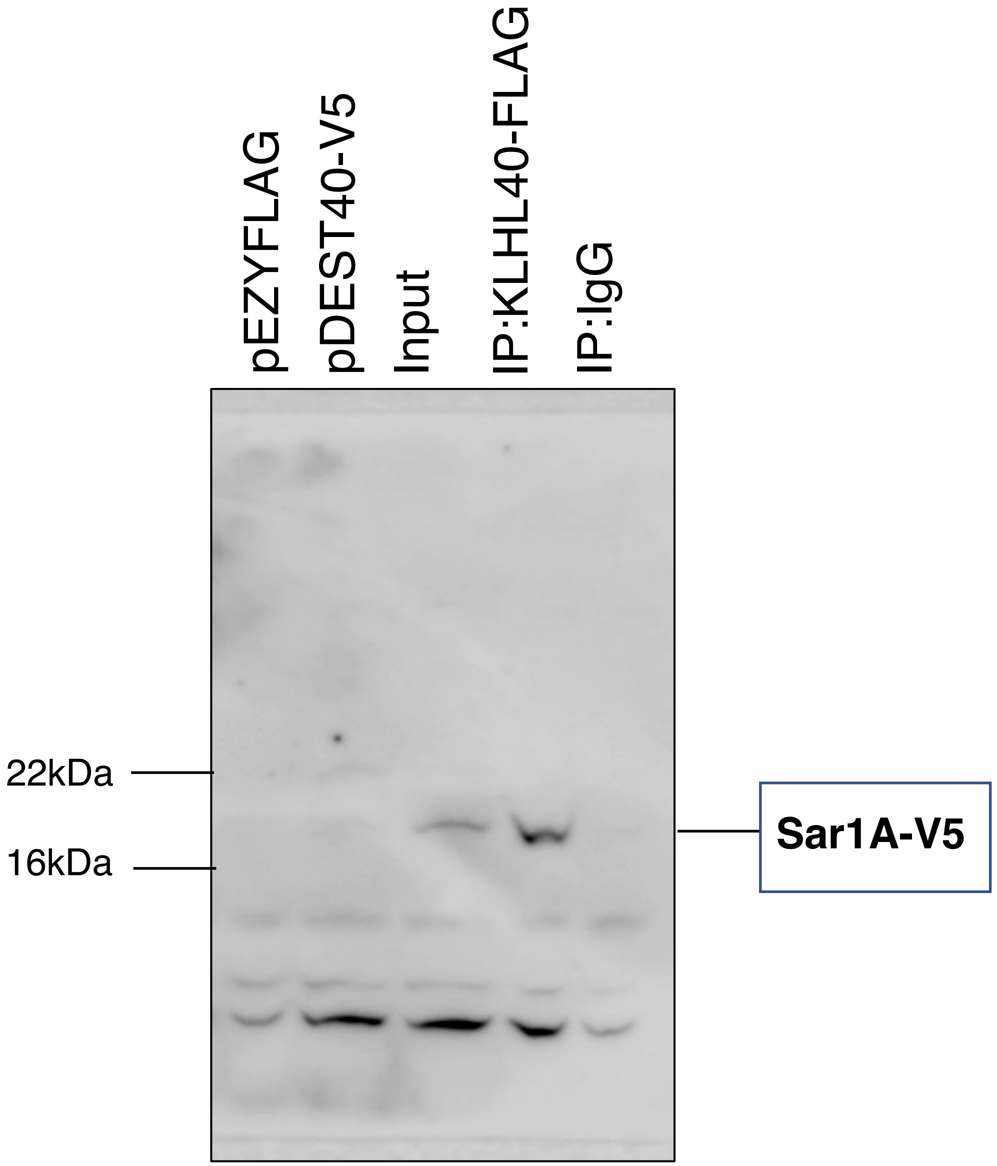

WB: Anti-V5: R960-25, Thermofisher Scientific

Figure 7B

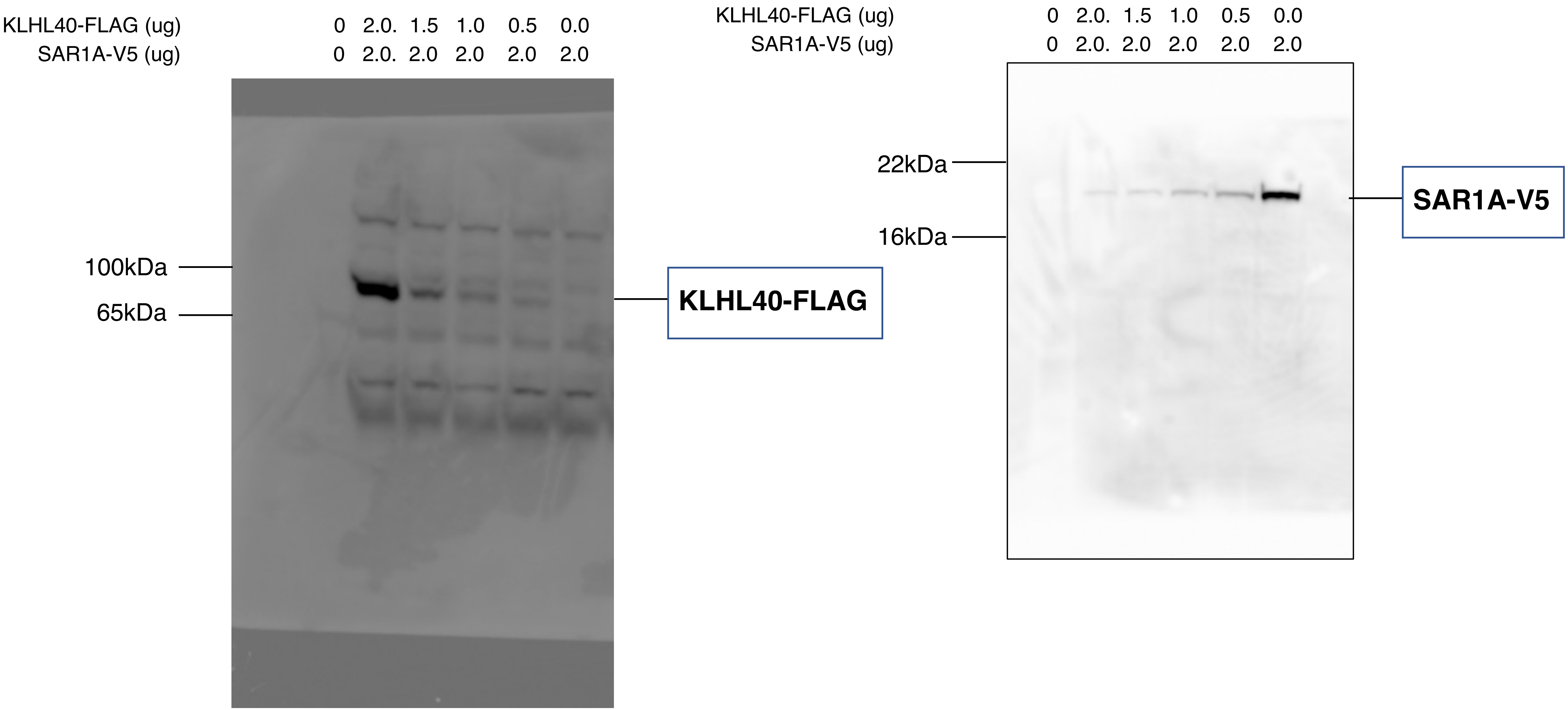

WB: Anti-FLAG: F1804, Sigma-Aldrich

WB: Anti-V5: R960-25, Thermofisher Scientific

Figure 7B

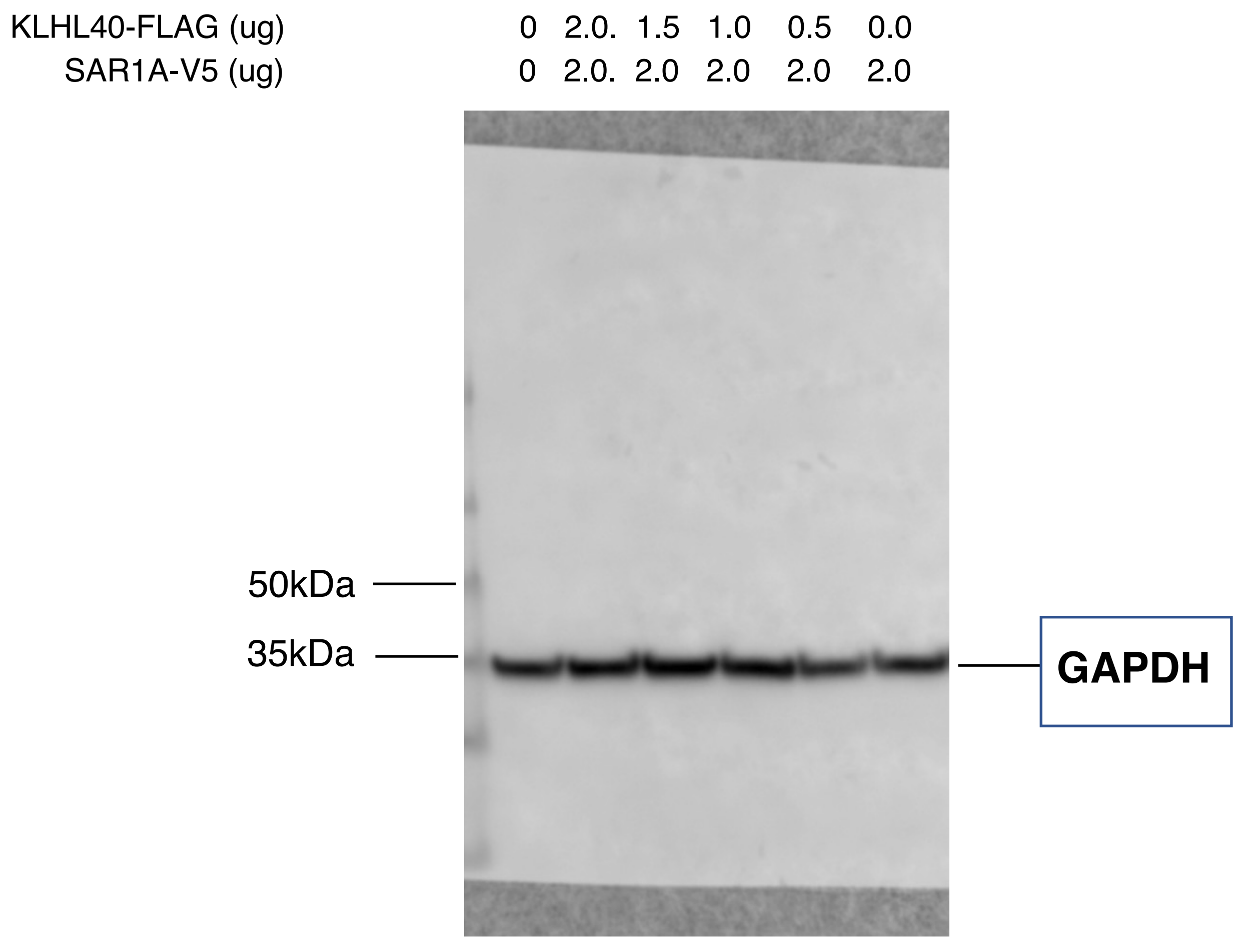

WB: Anti-GAPDH: ab8245, Abcam

Figure 7C

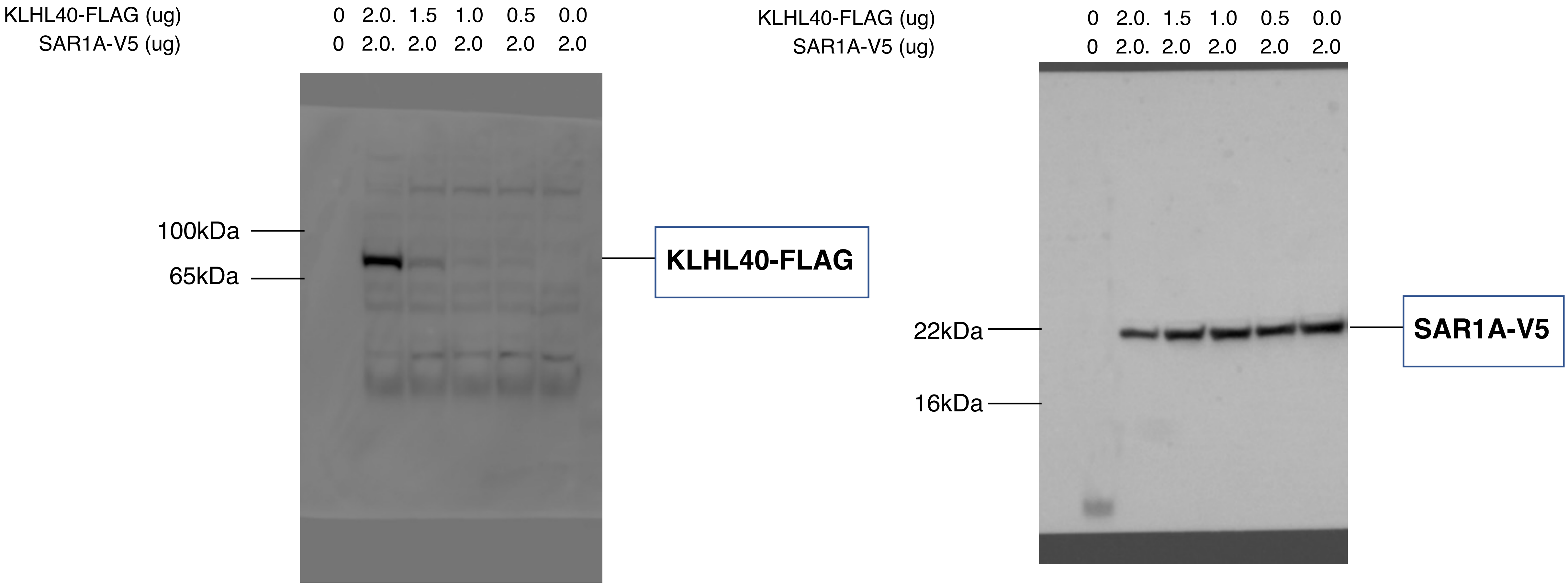

WB: Anti-FLAG: F1804, Sigma-Aldrich

WB: Anti-V5: R960-25, Thermofisher Scientific

Figure 7C

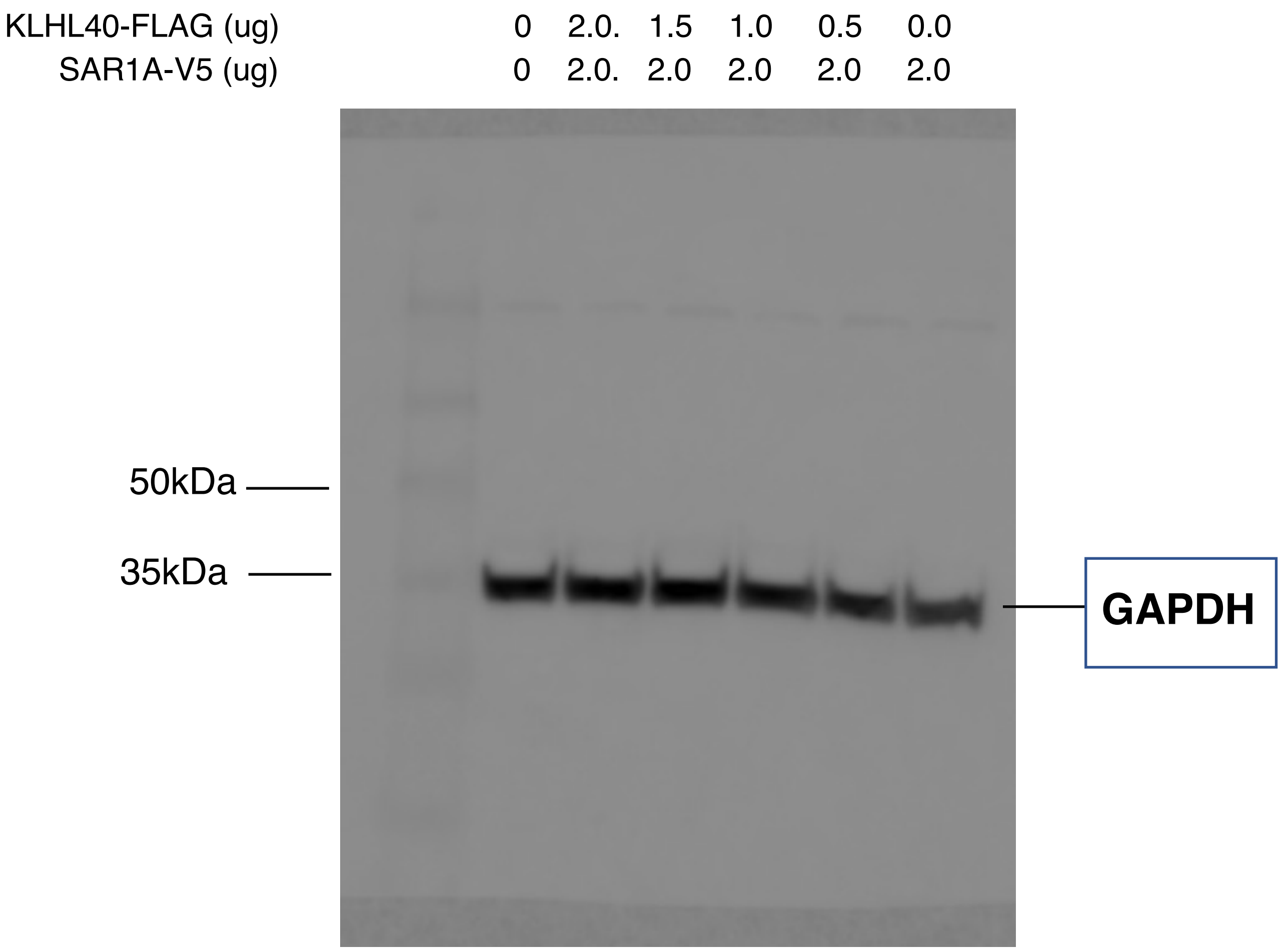

WB: Anti-GAPDH: ab8245, Abcam

Figure 7F

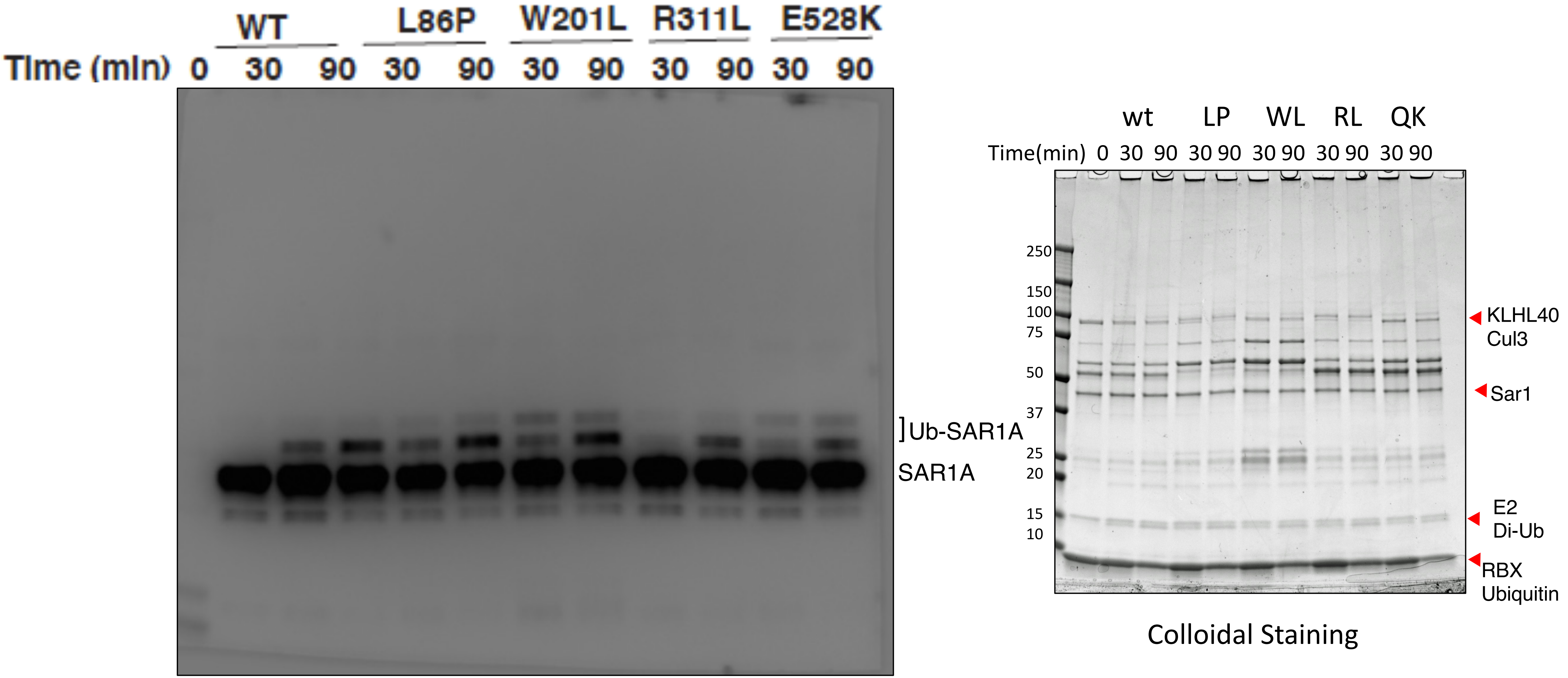

WB: Anti-Sar1: ab125871, Abcam

Colloidal Staining

Figure 7H

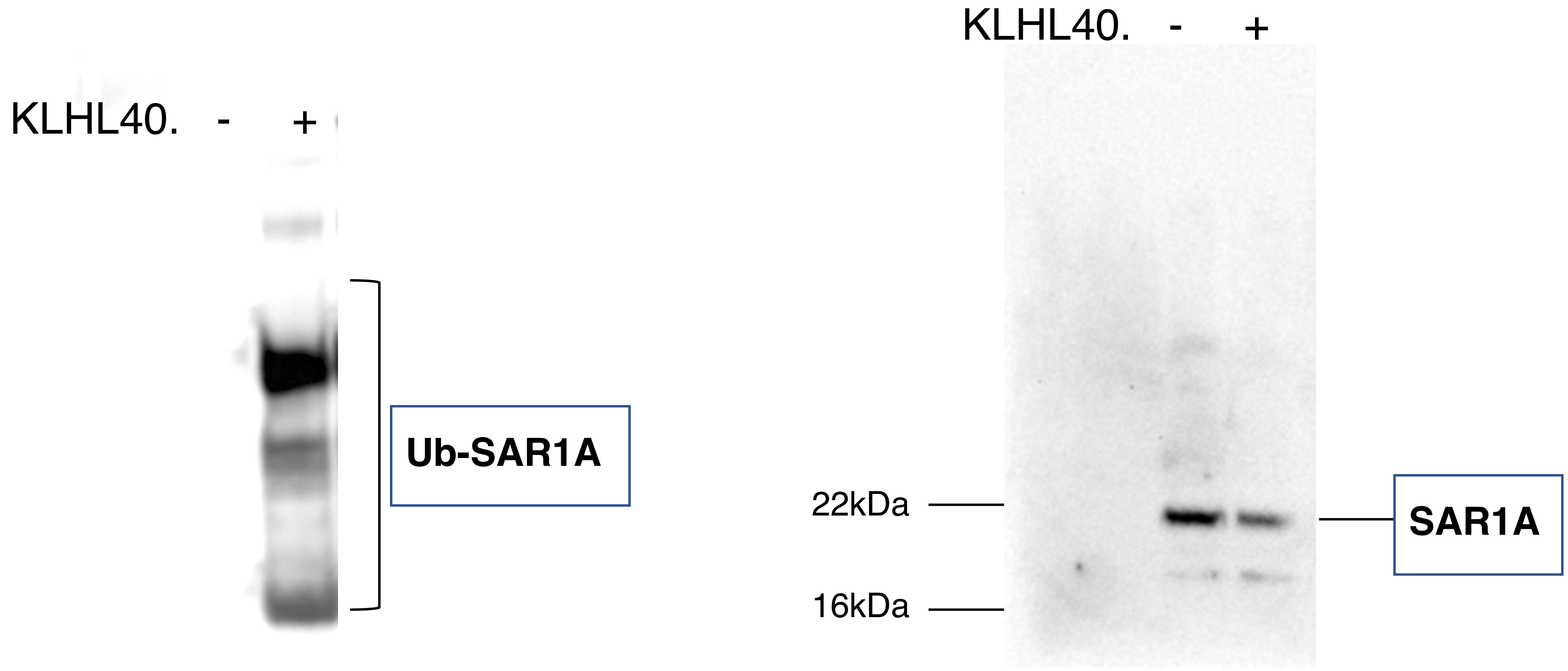

WB: Anti-Ubiquitin: ab7780, Abcam

WB: Anti-Sar1: ab125871, Abcam
