# Supplementary material for: Dynamic regulation of inter-organelle communication by ubiquitylation controls skeletal muscle development and disease onset
Source: eLife. 2023 Jul 11;12:e81966. doi: 10.7554/eLife.81966 (PMC10356137; doi:10.7554/eLife.81966)
Supplement: Supplementary file 10. [file elife-81966-supp10.docx]

Table S10: Sequences of the sgRNA target sites and primers sequences (5’-3’) to clone sgRNAs for creating *klhl40* zebrafish lines.

| **Gene** | **sgRNA Target site** | **Primers** |
| --- | --- | --- |
| *klhl40a-exon1* | GGACATCGAACCTGGCGTCA | F: TAGGACATCGAACCTGGCGTCA  R: AAACTGACGCCAGGTTCGATGT |
| *klhl40a-exon2* | GGCTGAGAACTCCATCTATG | F: TAGGCTGAGAACTCCATCTATG  R: AAACCATAGATGGAGTTCTCAG |
| *klhl40b-exon1* | GGACTGCATCAGGCTACG | F: TAGGACTGCATCAGGCTACGTC  R: AAACGACGTAGCCTGATGCAGT |
| *klhl40b-exon5* | GGACCGCAGTTCTCTCAGTC | F: TAGGACCGCAGTTCTCTCAGTC  R: AAACGACTGAGAGAACTGCGGT |
